# Effect of coronavirus lockdowns on the ambient seismic noise levels in Gujarat, northwest India

**DOI:** 10.1038/s41598-021-86557-9

**Published:** 2021-03-30

**Authors:** Ketan Singha Roy, Jyoti Sharma, Santosh Kumar, M. Ravi Kumar

**Affiliations:** 1 Institute of Seismological Research, Gandhinagar, 382009 India; 2 National Geophysical Research Institute, Hyderabad, 500007 India

**Keywords:** Seismology, Geophysics

## Abstract

The Covid-19 pandemic created havoc and forced lockdowns in almost all the countries worldwide, to inhibit social spreading. In India as well, as a precautionary measure, complete and partial lockdowns were announced in phases during March 25 to May 31, 2020. The restricted human activities led to a drastic reduction in seismic background noise in the high frequency range of 1–20 Hz, representative of cultural noise. In this study, we analyse the effect of anthropogenic activity on the Earth vibrations, utilizing ambient noise recorded at twelve broadband seismographs installed in different environmental and geological conditions in Gujarat. We find that the lockdowns caused 1–19 dB decrease in seismic noise levels. The impact of restricted anthropogenic activities is predominantly visible during the daytime in urban areas, in the vicinity of industries and/or highways. A 27–79% reduction in seismic noise ground displacement (d_rms_) is observed in daytime during the lockdown, in populated areas. However, data from station MOR reveals a drastic decrease in d_rms_ amplitude both during the day (79%) and night times (87%) since factories in this area operate round the clock. The noise at stations located in remote areas and that due to microseisms, shows negligible variation.

## Introduction

On March 24, 2020 at 8 p.m. (IST), as a preventive measure against the Covid-19 pandemic, the Indian government announced a complete lockdown of the whole country for 21 days starting from zero hour of March 25 (lockdown 1: March 25 to April 14, 2020), inhibiting movement of the entire 1.3 billion population of India, except emergency services. The lockdown was further extended for 19 days (lockdown 2: April 15 to May 3, 2020) permitting limited human activities from April 20, in specific regions where the spread had been contained or found minimal. The lockdown was further extended in phases till May 31, 2020 with restricted public and transport movement. Prior to the complete lockdown announcement, the Indian government had imposed a daytime (7 a.m.–9 p.m.) Janta Curfew (People’s Curfew) on March 22 (Sunday), restricting all kinds of human activities, as a trial measure (preparatory phase). However, from June 01, 2020, most of the human activities and economic services were resumed (Unlock), except in the containment zones, where the number of cases is high. The outbreak of the novel Covid-19 pandemic resulted in positive and negative consequences worldwide. While the lockdowns brought chaos to lives and devastated booming economies, many positive environmental changes are reported globally. These include drastic improvement in air quality leading to a clear panorama of magnificent landscapes from far distances, enhanced water quality in rivers, reduction in greenhouse gases and atmospheric temperature, sighting of exotic and wild animals in areas which were deserted by humans, and many more. Besides this, reduction in human-induced seismic noise is reported worldwide (e.g., Belgium, Italy) owing to measures that include closure of public places and travel restrictions^1–3^. Hence, the Earth’s buzz indicative of small vibrations underneath became a whisper due to restricted anthropogenic activities during the lockdowns. The lockdown conditions acted as a boon to seismologists in view of the sharp reduction in seismic background noise in the frequency range of 1 to 20 Hz, which corresponds to anthropogenic or cultural noise. This is primarily associated with lessened human activities, namely train and road traffic, and operation of machinery in factories.

Seismic background noise comprises ambient vibrations, cultural vibrations (from industry, traffic, etc.), temperature and atmospheric pressure fluctuations, effects of gravity and self-noise of the seismometers, recorded by broadband seismographs in a wide frequency range, from a few milli Hertz to several tens of Hertz^4, 5^. This noise is widely used for characterization of the source of origin (e.g., microseisms, blasts, migration of fluids), determination of crustal structure using surface waves extracted from cross-correlation, horizontal to vertical spectral ratios for estimating site effects in site specific hazard studies, apart from a wide range of other studies. These studies are performed in different frequency bands based on the purpose and source of origin (ambient environmental or anthropogenic processes). In general, natural ambient noise predominantly consists of the Earth’s hum and the microseism originating from the oceans^4, 6^. The hum constitutes continuously excited free oscillations of the Earth in the range of 2–20 MHz while the microseisms are dominantly Rayleigh waves observed between 0.04 and 1 Hz. However, earthquakes, which are mainly considered as sources in seismological studies, generate signals in the frequency range 3 × 10^–4^ to 20 Hz (0.05–3000 s), including body waves in 0.1–1 Hz and surface waves in 0.003–0.3-Hz range^7^. Seismic waves in the higher frequency range of 20–80 Hz are representative of explosions or other artificial sources, and used in shallow crustal studies. The short-period seismic noise may also be attributed to natural causes like wind, wind friction over a rough terrain, trees, and waterfalls, and/or man-made noise in the frequency range of about 0.5 Hz to ~ 15–60 Hz^8^. However, the dominant sources are man-made through machines, traffic, etc. Hence, short-period and short-term noise measurements are sufficient to characterize the high-frequency (f > 0.3 Hz) background noise and assess the potential influence of various types of man-made noise sources.

At short periods (1–50 Hz), noise from different directions due to various stationary and/or non-stationary random sources, gets superimposed to form a complex noise field and particle motion, in comparison to the long-period ocean noise. To avoid such complexities, seismometers are usually installed at isolated places, away from the cultural or anthropogenic noise sources (e.g., factories, highway, etc.), in order to record clean seismic signals. Therefore, selection of such sites is not always possible in city areas which are vulnerable to risks and important for seismic hazards studies. Since the quality of data is important in seismic studies, interference of useful signals with anthropogenic noise leads to loss of precious information, in view of the difficulty in isolating seismic signals from background noise covering the same frequency range. The variations in seismic noise levels have also been observed by analyzing data of day and night times, weekend and working days, and those in remote areas and cities^9, 10^. Further, observations in the frequency range encompassing anthropogenic noise are very useful for monitoring and tracking the causative sources^11^.

The lockdown measures taken worldwide led to a reduction in the seismic background noise, enabling better detection of microearthquakes using surface seismometers^1, 3^. Similarly, during the lockdown in India, many microearthquakes in the magnitude range of 0.6 to 1.5 have been detected in the seismically active Kutch seismic zone (KSZ), Gujarat, using data recorded by the Institute of Seismological Research (ISR) seismic network^12^, as shown in supplementary Figures S1 and S2. Moreover, in Surendranagar region of Saurashtra, an interesting pattern of no or few seismic events during the lockdown period (Fig. S3 in supplementary material), affirms ISR’s claims of quarry blast activity^12^. Thus, the cultural noise reduction due to the lockdown provided a rare opportunity to investigate the effect of anthropogenic noise on the seismic signals in terms of frequency and amplitude.

**Figure 1:**
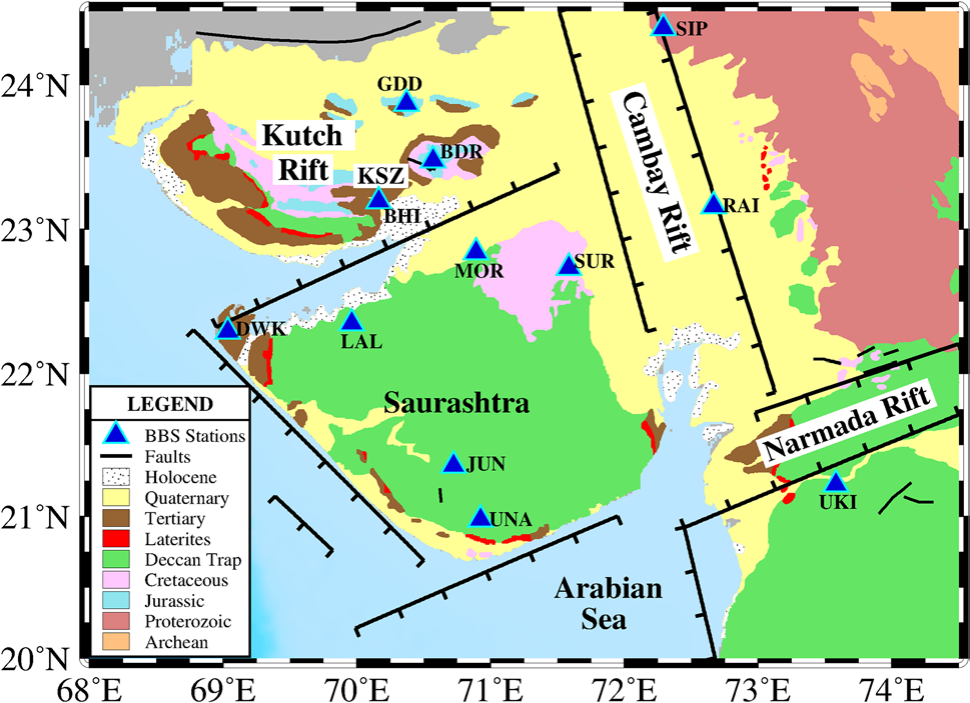
The distribution of 12 selected BBS stations superimposed on the geological map of Gujarat, northwest India. The major geographical regions are also marked. The Kutch, Cambay and Narmada rift basins are indicated using thick black lines. This figure is prepared using PyGMT (v0.3.0; https://www.pygmt.org/v0.3.0/)^24^.

In the present study, the variation in seismic background noise is estimated by calculating the power spectral density (PSD) and ground displacement, before and during the lockdown, using continuous seismic recordings at 12 online broadband seismograph (BBS) stations of the ISR seismic network (Fig. 1). The selected stations are sited on diverse geological and tectonic terrains in remote and city areas of Gujarat. Our analysis includes hourly and daily noise variations in different frequency ranges. The seismic noise variations are also compared for diurnal changes (day versus night), days of the week (working days versus weekends), public holidays and different subsurface geology.

## Data and methodology

The Gujarat State Seismic Network (GSNet), operated and maintained by ISR since July 2006, comprises a dense network of 60 digital BBS and 54 digital strong motion accelerographs (SMA) in the seismically active northwestern region of India^12^. Out of these, 49 permanent seismic stations, equipped with 120 s triaxial broadband sensors connected to 24-bit digitizers, record data at 50 samples per second (sps), and transmit in real time to the data center in ISR, through very small aperture terminal (VSAT) transmission. The other BBS stations are offline, recording data continuously at 100 sps^13^. The GSNet was initiated in the aftermath of the disastrous 2001 Bhuj earthquake (Mw 7.7), to monitor seismic activity. This was strengthened in a phased manner with the addition of BBS and SMA stations to the original network of 22 BBS and 43 SMA^12, 13^. At present, 25 broadband seismographs (BBS) are operating in a continuous mode in the seismically active zone of Kutch region (popularly called as Kutch Seismic Zone, KSZ), and 10 of these stations are located at an epicentral distance of ~ 50 km from the 2001 Bhuj earthquake (Figure S1). All stations are located in the same environmental conditions and have the same technical specifications^12–14^.

The quality of seismic stations is generally evaluated using power spectral density (PSD) estimates of their records^3^. To estimate the power spectral densities (PSDs), Welch’s technique^3, 15^ is applied to the 30-min-long noise segments of the continuous signal with a 50% overlapping window, using Obspy^16, 17^. The purpose of using overlapping time series segments is to reduce the variance in the PSD estimates^18^. The estimated PSDs are represented in decibels (dB) with respect to acceleration (meters^2^/second^4^)/Hertz, in the frequency domain. Prior to the PSD analysis, all the data were corrected for instrument response and baseline during the initial pre-processing. However, to understand the true variation of noise at a given station, calculation of probability density functions (PDFs) from the estimated PSDs for the selected time period is important^18^. Hence, PDFs were estimated to characterize the variation of PSDs at the 12 selected BBS stations for the pre-lockdown (March 01–24, 2020) and co-lockdown (March 25–April 19, 2020 except for station RAI) periods using continuous recordings of seismic signals (supplementary Fig. S4). The lockdown duration for station RAI is March 25–May 17, 2020. We have also computed and plotted the mean, median and mode powers for each period bin in the frequency range of 0.1 to 20 Hz and represented in supplementary Figure S4. We have not observed any major difference in the estimated mean, median and mode for the pre- and co-lockdown durations. However, the median or mode is preferred over mean in PSD-PDFs estimates of seismic noise, to exclude large outliers in the data, e.g. earthquakes, system transients, instrumental glitches, calibration pulses^18^. Hence, in the present study, we preferred to consider median over mode since the latter is often affected by system transients (e.g. telemetry dropouts)^18^. However, we have removed the local earthquakes from the datasets for the studied duration, taking into cognizance the earthquake catalogue compiled by ISR datacenter. Here, we have analysed and discussed the PSDs with respect to acceleration, since most of the previous seismic background studies presented the PSD estimates with respect to acceleration^9, 18^. However, we have provided the PSD variation with respect to acceleration, velocity and displacement^10^ in supplementary figure S5. To monitor the anthropogenic changes, we preferred to monitor day-wise changes in ground displacement. Hence, to compute the seismic noise level in ground displacement, the estimated PSD in acceleration in dB (i.e., $P_{a} [dB] = 10\log_{10} [P_{a} /1*(m/s^{2} )^{2} /Hz]$) is then converted into displacement spectral power (Disp_*pow*_) using the following relation, $Disp_{pow} = {{10^{{\left( {{{P_{a} [dB]} \mathord{\left/ {\vphantom {{P_{a} [dB]} {10}}} \right. \kern-\nulldelimiterspace} {10}}} \right)}} } \mathord{\left/ {\vphantom {{10^{{\left( {{{P_{a} [dB]} \mathord{\left/ {\vphantom {{P_{a} [dB]} {10}}} \right. \kern-\nulldelimiterspace} {10}}} \right)}} } {(2\pi f)^{2} }}} \right. \kern-\nulldelimiterspace} {(2\pi f)^{2} }}$^3, 19^. Later, the root mean squared (rms) displacement (d_rms_) is estimated in the time domain by applying a bandpass filter to Disp_pow_ in the selected frequency band, using Parseval’s identity, $d_{rms} (t) = \sqrt {\int\limits_{{f_{\min } }}^{{f_{\max } }} {Disp_{pow} (f)df} }$. We have also studied the variation in seismic noise levels using d_rms_ values in different frequency bands between 1 and 20 Hz, to analyze the effect of anthropogenic noise during pre-, co- and post-lockdown periods. The median of the estimated d_rms_ values is considered for each calendar day. Using d_rms_ values, the diurnal variations are also studied in different frequency bands between 1 and 20 Hz. Here, daytime is considered as 7 a.m. to 7 p.m. (07–19 h) in Indian standard time (IST) and the nighttime as 10 p.m. to 5 a.m. (22–05 h) in accordance with traffic and human activities.

In Gujarat, most of the human activities resumed from April 20, 2020 onwards, except in six major districts (including Gandhinagar where station RAI is located), which were heavily affected by Covid-19. However, after May 31, 2020 most of the cultural activities resumed in a phased manner with some limitations, and named as Unlockdown series for different durations. Hence, we have also analyzed the variation in d_rms_ values for the Unlockdown period (Unlockdown 1: June 01–10, 2020) at the considered stations to observe the steep increase in the noise levels in the anthropogenic frequency ranges.

## Seismic noise characterization

**Figure 2:**
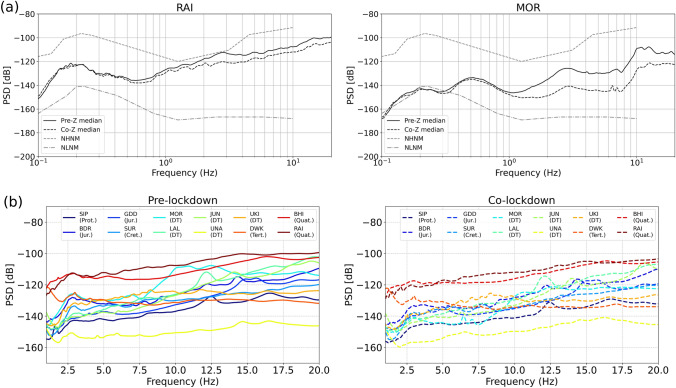
Seismic background noise levels with respect to frequency before and during the lockdown in terms of PSD using median values. (**a**) The variation in PSD value in terms of median for the vertical (Z) component is shown for the pre- and co-lockdown periods in the frequency range of 0.1–20 Hz, that is representative of ambient and anthropogenic noise for two different stations (MOR, RAI) of ISR seismic network. PSD variation for other stations is shown in supplementary Figure S6. The new high and new low noise models (NHNM, NLNM)^9^ are represented by gray lines, as upper and lower limits. (**b**) Comparison of PSD in the high frequency range of 1–20 Hz (representative of anthropogenic noise sources) for pre- and co-lockdown periods at all the studied stations sited on varied geological formations. The geological formations at different stations are Proterozoic rocks (SIP), Jurassic rocks (BDR, GDD), Cretaceous rocks (SUR), Deccan Traps (MOR, LAL, JUN, UNA, UKI), Tertiary sediments (DWK), and Quaternary sediments (BHI, RAI). Local anthropogenic conditions at these stations are mentioned in Table 1. Matplotlib is used during preparation of Figs. 2, 3, 4, 5, 6, 7 using Python in Jupyter notebook^25–27^.

**Figure 3:**
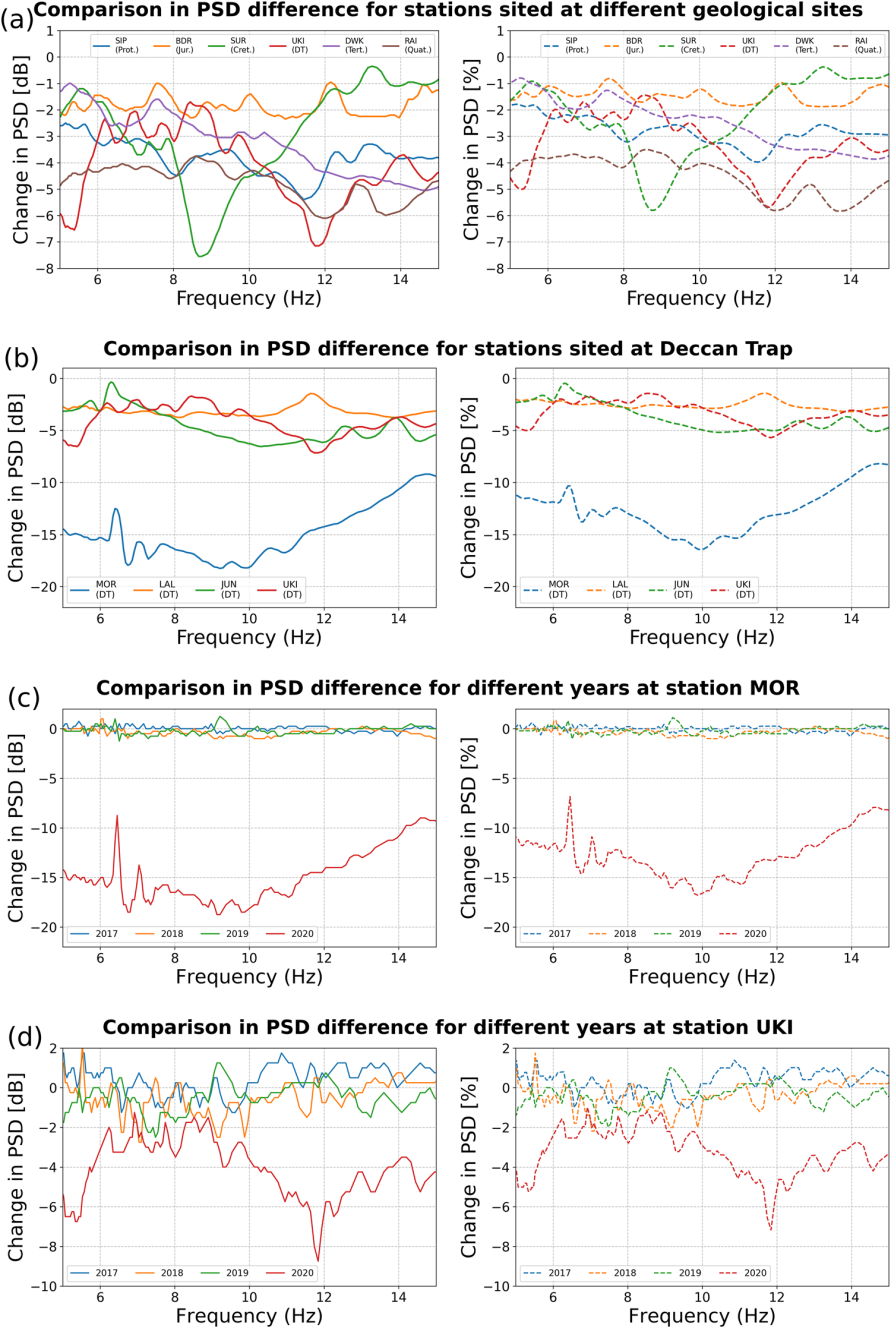
(**a**,**b**) The change in PSD values during co-lockdown period with respect to the pre-lockdown for different stations sited on varied and same geological formations. Here, change in PSD [dB] = $median(PSD)_{co - lockdown} - median(PSD)_{pre - lockdown}$, and change in PSD [%] = $(median(PSD)_{co - lockdown} - median(PSD)_{pre - lockdown} )*100/median(PSD)_{pre - lockdown}$. (**c**,**d**) The comparison in change in PSD values for different years for the same duration as in the pre- and co-lockdown periods are shown for stations MOR and UKI. The change in PSD values are represented in dB and percentage considering median values in the frequency range of 5–15 Hz, representative of anthropogenic noise sources. The subsurface geology and other local anthropogenic conditions are mentioned in Table 1.

The impact of lockdown is predominantly visible in a wide range of frequencies above 1 Hz (representative of anthropogenic noise), as shown in Figs. 2 and S6 in supplementary material. However, as expected, no or negligible variation is observed in the ambient noise due to microseism sources, at frequencies below 1 Hz, in the pre- and co-lockdown periods (Fig. 2a). The PSDs are estimated for all the vertical components and compared with the new high and new low noise models (NHNM, NLNM)^9^. The comparison of PSDs for different types of subsurface geological formations for pre- and co-lockdown duration is shown in Figs. 2b, 3 and listed in Table 1. In Fig. 2b, the variation in PSD median estimates is shown for all the stations in the high frequency range of 1–20 Hz, representative of anthropogenic noise sources for the pre- and co-lockdown duration. Here, we can clearly observe a large variation in seismic noise levels for different types of subsurface geology. A remotely located station like UNA, atop the basaltic Deccan Traps has the minimum seismic background noise amplitude (Fig. 2b). Similarly, station RAI overlying Quaternary sediments shows a large amplitude variation compared to stations sited on hard rocks (Figs. 2b and 3a). Previously, large seismic background noise values in the 2–5 Hz frequency range for stations located on the Quaternary and Tertiary formations were reported^20^, and explained in terms of a combined effect of predominant frequencies and cultural activities. Here, the predominant frequency corresponds to the frequency at which the maximum value of site amplification is observed for a station/site.

**Table 1:** Variation in the seismic noise level (in percentage) at different stations during the day and night times in the lockdown period, using the ground displacement values for different frequency bands between 1 and 20 Hz.

Stations	Geology	Time	Change in seismic noise level during lockdown using median displacement values of d_rms_ (in %)					Change in PSD during lockdown in 5–15 Hz		Other information
			1–5 Hz	5–10 Hz	10–15 Hz	5–15 Hz	15–20 Hz	(Range), median (dB)	Median (%)	
BHI	Quaternary sediments	Day	− 40	− 32	− 26	− 30	− 29	(− 2, − 4), − 2	− 2	Located at the rural community hall
		Night	− 38	− 32	− 30	− 31	− 37			
RAI	Quaternary sediments	Day	− 51	− 43	− 49	− 45	− 44	(− 4, − 6), − 5	− 4	Located in ISR campus
		Night	− 44	− 28	− 22	− 25	− 1			
DWK	Tertiary sediments	Day	− 10	− 26	− 51	− 31	− 46	(− 1, − 5), − 3	− 2	Located in a coastal region in the vicinity of a famous religious place
		Night	− 2	− 1	− 12	− 2	0			
MOR	Deccan traps	Day	− 75	− 83	− 78	− 79	− 58	(− 9, − 19), − 16	− 13	Located in the city, surrounded by Factories (run 24 × 7) and main road
		Night	− 66	− 87	− 87	− 87	− 70			
JUN	Deccan traps	Day	− 15	− 39	− 49	− 48	− 39	(0, − 7), − 5	− 4	Located in the vicinity of main road
		Night	− 8	− 13	− 36	− 29	26			
LAL	Deccan traps	Day	− 22	− 32	− 26	− 27	− 34	(− 1, − 4), − 3	− 3	Located in the city near to highway
		Night	− 12	− 39	− 30	− 32	− 43			
UKI	Deccan traps	Day	− 43	− 37	− 49	− 39	− 36	(− 1, − 9), − 4	− 3	Located in the city near to road
		Night	− 29	− 18	− 43	− 21	− 36			
UNA	Deccan traps	Day	− 5	− 16	− 2	− 7	14	(3, − 1), 1	1	Located in a remote area in isolated place
		Night	− 9	38	34	36	47			
SUR	Cretaceous rocks	Day	− 29	− 31	− 22	− 29	− 9	(0, − 8), − 3	− 2	Located in a remote area near to check dam, less populated
		Night	− 27	− 32	− 23	− 30	5			
BDR	Jurassic rocks	Day	− 30	− 35	− 31	− 35	− 17	(0, − 3), − 2	− 2	Located in a remote area, less populated
		Night	− 42	− 8	− 2	− 5	− 8			
GDD	Jurassic rocks	Day	0	− 4	− 34	− 18	− 29	(1, − 4), − 1	− 1	Located in a remote area, in the vicinity of circuit (guest) house
		Night	− 8	10	19	12	54			
SIP	Proterozoic rocks	Day	− 42	− 34	− 34	− 34	− 29	(− 3, − 6), − 3	− 3	Located in a remote area, near to dam, less populated
		Night	− 36	− 42	− 46	− 45	− 43			

Here, the difference during pre- and co-lockdown periods is estimated by considering the median values for the estimated periods. Negative values indicate a drop in the seismic noise level (in %) during co-lockdown period with respect to the pre-lockdown, i.e., $(median(d_{rms} )_{co - lockdown} - median(d_{rms} )_{pre - lockdown} )*100/median(d_{rms} )_{pre - lockdown}$.Positive values represent increase in the anthropogenic noise during the lockdown period. The changes in PSD are also calculated using median values for the pre- and co-lockdown periods. The range of change in PSD (in dB) in the 5–15 Hz frequency range during lockdown with respect to pre-lockdown is also listed. Here, the amplitude of PSD difference represents the absolute change and negative sign indicates a drop in PSD amplitude during the co-lockdown with reference to the pre-lockdown. The median values for the change in PSD (in dB and %) are also provided. The subsurface geology at different stations and other local conditions are also illustrated. Here, change in PSD [dB] = $median(PSD)_{co - lockdown} - median(PSD)_{pre - lockdown}$, and change in PSD [%] = $(median(PSD)_{co - lockdown} - median(PSD)_{pre - lockdown} )*100/median(PSD)_{pre - lockdown}$. During calculation, the pre-lockdown duration is considered from 01/03/2020 to 24/03/2020 and co-lockdown from 25/03/2020 to 19/04/2020 for the stations, except for RAI. For station RAI, co-lockdown duration is 25/03/2020 to 17/05/2020.

The station MOR, located on the Deccan Traps, shows large seismic noise amplitudes compared to other stations on the Deccan Traps, in the frequency range of 5–15 Hz (Fig. 3b and Table 1). A striking reduction of 9–19 dB in noise amplitude is observed at MOR, during the lockdown, due to the closure of ceramic factories. A large variation is observed in the range of ~ 9–11 Hz, which is reported as the predominant frequency for station MOR, with an amplification of ~ 6 in this frequency range^21^. Contrary to this, other stations located on Deccan Traps (subsurface geology) having different local cultural conditions in comparison to MOR reveal a lesser amount of seismic background noise reduction (Fig. 3b). Here, we chose 5–15 Hz, since in this frequency range, the effect of local earthquakes and wind-induced seismic waves are minimal^10, 22^. Moreover, seismic noise studies worldwide considered ~ 5–15 Hz for representing the effect of anthropogenic noise reduction during the coronavirus lockdowns^3, 23^. Other stations also reveal a reduction in seismic noise amplitude in terms of PSD value (change in median PSD values: 1–16 dB and 1–13%) during the lockdown period (Table 1), except at UNA. At station UNA, an increase in PSD values during the lockdown period is observed, which might be representative of some kind of anthropogenic activities (e.g., emergency services allowed by local authorities). The behavior at the studied stations reflects a contribution of subsurface geology and cultural noise in terms of amplitude and frequency (Figs. 2, 3 and Table 1). A comparison in the change in PSD values during the lockdown period is also made with respect to the previous years (2017–2019) for stations MOR and UKI, by taking exactly the same duration considered for pre- and co-lockdown periods (Figs. 3c,d). Here, we clearly observe the effect of anthropogenic noise sources on the seismic noise level.

**Figure 4:**
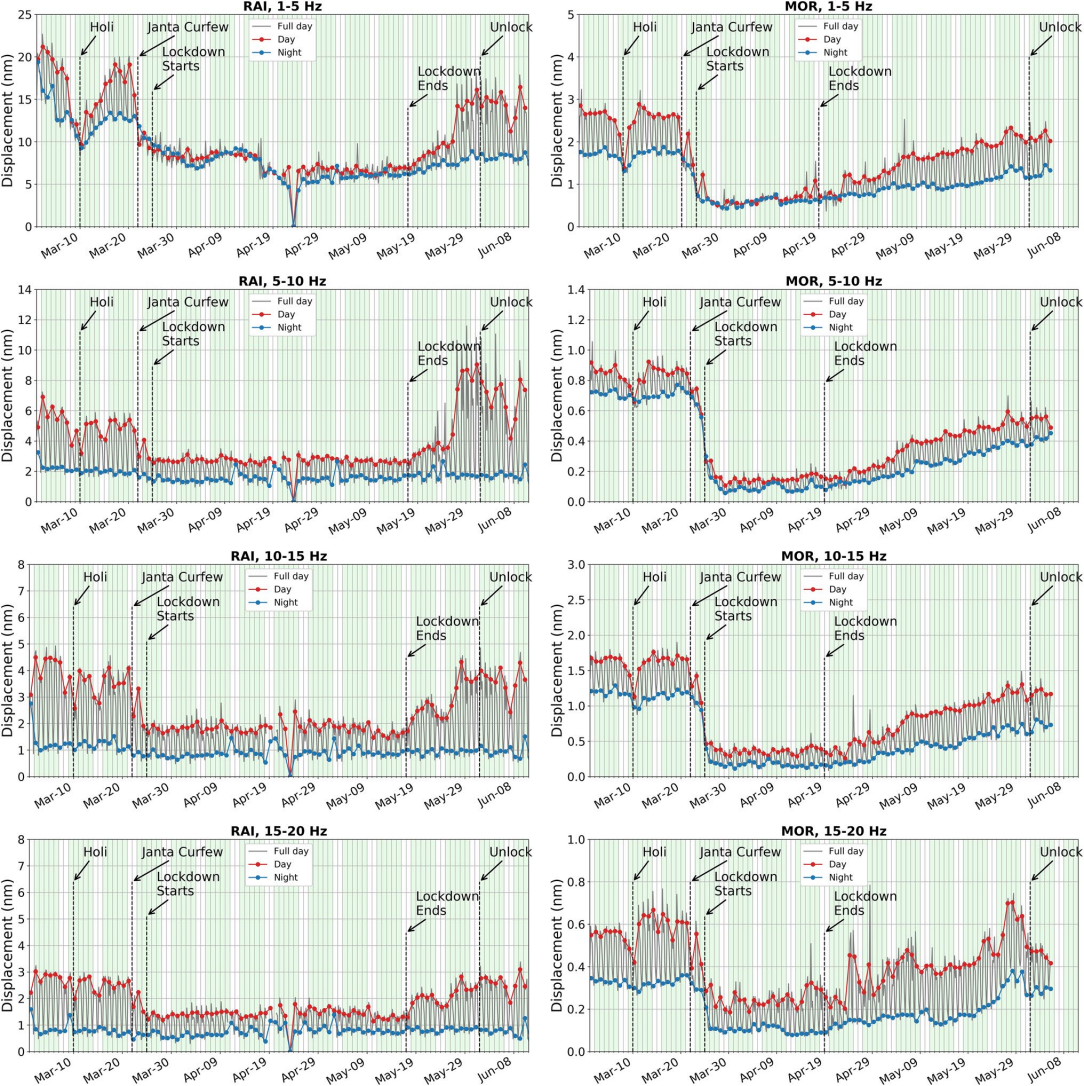
Day wise ground displacement variation in terms of median d_rms_ for the pre-, co- and post-lockdown periods at stations MOR and RAI in different high frequency ranges (1–5 Hz, 5–10 Hz, 10–15 Hz and 15–20 Hz) between 1 and 20 Hz. The variation in ground displacement during daytime and nighttime is also shown. The complete lockdown period, beginning of Unlock, Janta Curfew and extended holiday during Holi (Indian Festival) are also marked. Subsurface geology and other local anthropogenic conditions are mentioned in Table 1.

In the present study, the effect of anthropogenic activities on the ground displacement due to seismic background noise is significantly observed during pre-, co- and post-lockdown periods in different frequency ranges, 1–5 Hz, 5–10 Hz, 10–15 Hz, 15–20 Hz and 20–25 Hz, at all the considered stations. The results for example stations RAI and MOR in different frequency bands between 1 and 20 Hz are shown in Fig. 4 and those for other stations are provided in supplementary Figure S7. In the present study, we have not analysed the seismic noise recorded in the 20–25 Hz since the nyquist frequency for the data used is ~ 24–25 Hz. Besides this, the extent of fall in amplitudes in 20–25 Hz is of the same amplitude (or lesser) as observed in the 15–20 Hz. This is shown for stations RAI and MOR in supplementary Figure S8. Thus, the upper limit is set at 20 Hz. The variation in seismic noise is represented in terms of d_rms_, for each calendar day. The diurnal variations at the stations are also studied in the above mentioned frequency ranges. Matching with the anthropogenic activity in the pre-lockdown period during the day and night times, a similar pattern is observed in hourly variation of seismic noise in different anthropogenic frequency bands between 1 and 20 Hz, as shown for station RAI (supplementary Figure S9). However, the ground displacement plots in different frequency ranges, for the 12 stations, reveal that the noise amplitude is large in the 1–5 Hz range compared to that in 5–20 Hz, which might be the effect of subsurface geological strata, besides anthropogenic noise sources (Fig. 4 and supplementary Figure S7). Stations installed on sedimentary formations (Quaternary, Tertiary) show noise levels that are ~ 5–25 times larger in comparison to those on hard rocks (Deccan Traps, Proterozoic formations). The seismic noise amplitudes at stations sited on Deccan traps comprising tholeiitic basalts (e.g., MOR, JUN, and LAL) are predominant in the 1–5 Hz and 10–20 Hz range. Large values of seismic background noise correspond well with the predominant frequencies. The predominant frequencies for stations MOR, JUN and LAL are found between 6 and 10 Hz^21^. However, these results are limited up to 10 Hz. This is true for other stations on different geological formations as well. Moreover, the effect of amplification is negligible in hard rock sites compared to that in soft sediments.

**Figure 5:**
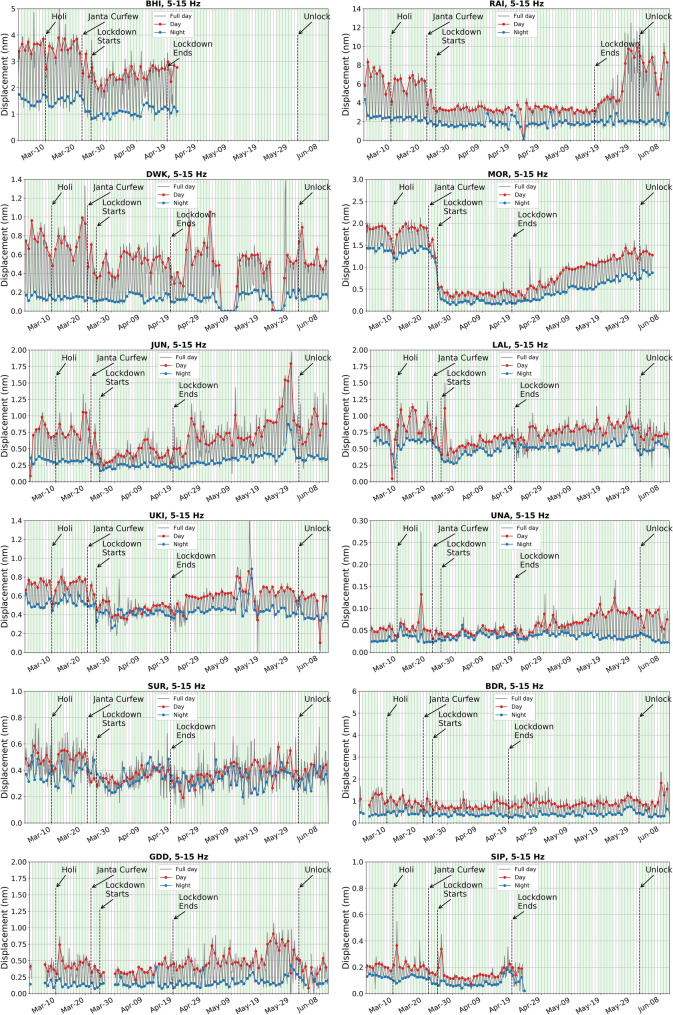
Day wise ground displacement variation in terms of median d_rms_ for the pre-, co- and post-lockdown periods in the frequency range of 5–15 Hz, at all the studied stations. The variation in ground displacement during daytime and nighttime is also shown. The complete lockdown period, beginning of Unlock, Janta Curfew and extended holiday during Holi (Indian Festival) are also marked. For stations BHI and SIP, data is not available after April 22, 2020 due to technical reasons. The lockdown ended on April 19, 2020 at these stations, hence we have considered these stations for analysis. Subsurface geology and other local anthropogenic conditions are mentioned in Table 1.

**Figure 6:**
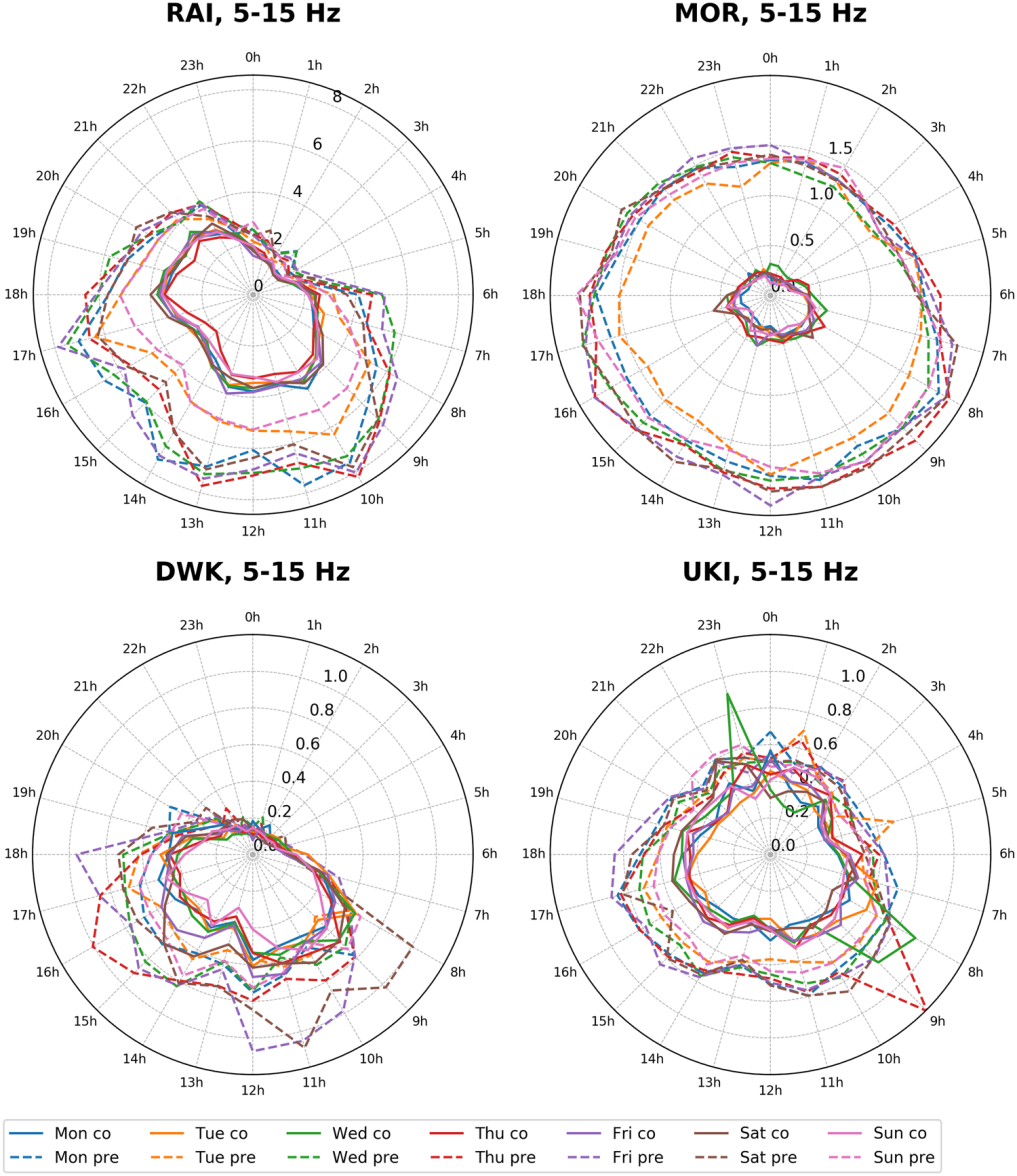
Hour wise variation in seismic background noise for different weekdays, in the frequency range of 5 to 15 Hz, in terms of ground displacement (d_rms_, in nm) for pre- and co-lockdown periods at RAI, MOR, DWK and UKI stations. The d_rms_ value on any particular day is representative of the median of all the corresponding days for pre- and co-lockdown periods. Here, time is mentioned in Indian Standard time (IST). Subsurface geology and other local anthropogenic conditions are mentioned in Table 1. Similar plots for other stations are provided in supplementary Figure S10.

**Figure 7:**
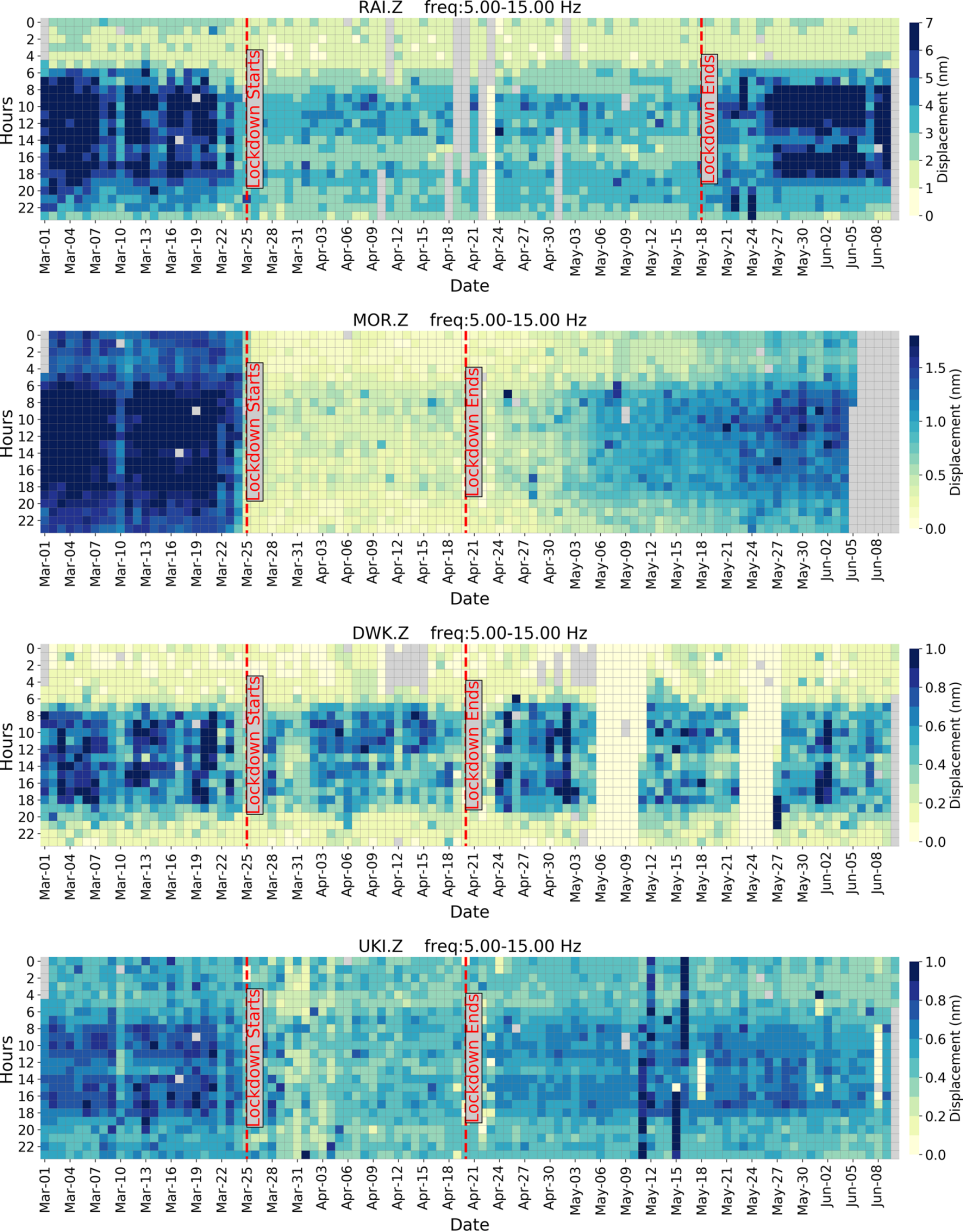
Day wise hourly variation in ground displacement (d_rms_, in nm) during pre-, co- and post-lockdown periods at RAI, MOR, DWK and UKI stations in the frequency range of 5–15 Hz. Here, the data are normalized to the 15–85th percentiles of the pre-lockdown period for better representation and comparison purpose. Gray colour represents the data gap. Time is mentioned in IST. Subsurface geology and other local anthropogenic conditions are mentioned in Table 1. Plots for other stations are shown in supplementary Figure S11.

On analyzing the seismic background noise through PSD and d_rms_ median values in different frequency ranges between 1 and 20 Hz, the effect of lockdown is prominently visible in the 5–15 Hz range, with minimal interference from local earthquakes and other environmental factors^3, 10, 22^. Besides this, in the frequency range of 5–15 Hz, the diurnal variation is quite evident and follows the same pattern during the pre- and co-lockdown periods, with lesser d_rms_ amplitude recorded in the latter (Fig. 5). Hence, in our case, we have considered the 5–15 Hz range to discuss diurnal and hourly variations at all the stations (Figs. 5, 6 and 7). Moreover, we have removed the records of local earthquakes from the datasets at all the stations. The hourly plots represent the median of d_rms_ on the corresponding days (Fig. 6). For example, the median value of all Mondays before the lockdown period is considered for the pre-lockdown plot and the median of all Mondays which fall during complete lockdown represent the co-lockdown plot. In Figs. 6 and 7 the results for specific stations (RAI, MOR, DWK and UKI) are shown, where major or some changes are observed between pre- and co-lockdown periods. Similar plots for the remaining stations are presented in supplementary Figures S10 and S11. The variation in d_rms_ amplitude (in %) at different stations during pre- and co-lockdown are also shown in different frequency bands between 1 and 20 Hz (Table 1), which provides a comprehensive picture of the effect of the coronavirus lockdown in terms of reduction in anthropogenic noise levels. The variation in PSD values in the 5–15 Hz frequency range due to complete lockdown is listed in terms of range of median value (in dB and %) in Table 1. The significance of the estimated PSD and d_rms_ values in different frequency ranges are provided for the pre- and co-lockdown periods in supplementary Tables S1–S3. Here, the confidence interval of the population mean for the PSD and d_rms_ values are provided with 95% confidence level. With the same confidence, the confidence interval of the difference in the population mean of the seismic noise level (PSD and d_rms_) between pre- and co-lockdown duration are also listed in supplementary Tables S1–S3.

## Discussions

We observed different trends in the seismic noise levels during pre-and co-lockdown periods based on the location of the stations. As expected, stations in or near big cities show a large drop in noise, whereas those in remote locations remain quiet as ever. We have considered the median d_rms_ values in the frequency range of 5–15 Hz to discuss the change in the anthropogenic seismic noise level during the lockdown period in the vicinity of the stations (Fig. 5; Table 1). During the lockdown, a sharp decrease in seismic noise is observed at station MOR, located in the vicinity of ceramic factories, due to the closure of all kinds of activities (Fig. 5; Table 1). Both during the day (79%) and nighttime (87%), we observed a sharp reduction in noise levels, since these factories work round the clock. Since machineries in these ceramic factories are operated throughout the year on all days round the clock, a large drop in d_rms_ values is noticed for all days of the week during the pre- and co-lockdown periods, for station MOR (Fig. 6). Further, during the pre-lockdown period, the noise levels peak during 7–9 h and 17–19 h at MOR, due to a change in workers shift, where a new batch of workers relieve those working in the previous shift (Fig. 6). Also, a main road runs in the vicinity of this station, where the traffic movement is at its peak during 7–9 h and 17–19 h. For a more clear image of the seismic noise level at different hours, the median d_rms_ values are provided for 24-h of every single day for pre- to post-lockdown duration (Fig. 7). The reduction in anthropogenic noise level is predominantly observed during the lockdown period, returning to previous levels after the lockdown, at all the stations (Fig. 7 and supplementary Figure S11). We have also correlated the change in the seismic noise pattern for station MOR with the traffic mobility data of Gujarat and find that they exactly replicate the trend during pre-, co- and post-lockdown periods (supplementary Figure S12). Similar to MOR, a sharp reduction in the median d_rms_ is observed for station RAI, which is located in the ISR campus building at Gandhinagar, the capital of Gujarat state (Figs. 4, 5 and 6). At this station, we observed a 45% and 25% reduction in the seismic noise level during the day and night, respectively (Table 1). The peaks at 10, 13 and 17 h in the 5–15 Hz frequency range, during the pre-lockdown, may represent the arrival of staff for office work, lunch time and departure timings respectively, when the human activity is maximum (Fig. 6). Also, stations LAL, DWK, JUN and UKI, located in the vicinity of highways, busy roads or public places reveal a 27–48% and 2–32% reduction in noise levels during the day and night times, respectively, in the 5–15 Hz frequency range (Fig. 5; Table 1). Similarly, station BHI in the vicinity of a school and community center in a less populated village of Kutch region, shows a 30% and 31% decrease in the median noise level during day- and night-time, respectively. However, the fall in noise levels is not significant at station BHI due to some ongoing activities that are part of precautionary health measures to cope with the Covid-19 pandemic. The stations BDR, GDD, and UNA located in remote areas, show less variation in the median d_rms_ (7–35%) for daytime in the frequency range of 5–15 Hz (Table 1). However, an increase in the seismic noise level is observed during the nighttime at stations GDD and UNA, by 12% and 36%, respectively. The increase in the median d_rms_ values during nighttime in the lockdown period might be a representative of some kind of ongoing anthropogenic activities at that time in the vicinity of the stations GDD and UNA (e.g., emergency services, authorized or unauthorized human activities). In summary, the noise levels in the frequency range of 5–15 Hz (mainly representative of anthropogenic noise), at stations located in the vicinity of factories and in populated areas reduced sharply due to complete lockdown from March 25 to April 19, 2020. However, after April 20, 2020, a gradual rise in seismic noise levels is markedly visible in the 5–15 Hz frequency range for all the seismic stations analysed, except RAI, due to the removal of lockdown and revival of human activities. In Gandhinagar region, where RAI station is located, the lockdown is continued till May 17. Hence, an increase in seismic noise level is observed after this day. A similar pattern of drop in seismic noise level (~ 10–50%) is observed at stations located in the city and industrial areas during the daytime on long weekends, public holidays, festival vacations (e.g., Holi on March 10, Tuesday preceded by weekend), and Janta Curfew (Fig. 5). However, the drop is not as drastic as it has been observed during complete lockdown. Concisely, we have observed a seismic noise reduction of 27–79% in urban populated areas, 29–35% in rural populated areas, and 7–18% at remotely located stations, in terms of median d_rms_ during the daytime when maximum anthropogenic activities take place in the 5–15 Hz frequency range (Table 1). A positive correlation between the seismic noise and anthropogenic mobility record provides a new dimension in real time monitoring using seismological datasets.

## Conclusions

In the present study, we have clearly estimated the reduction in seismic noise during the lockdown, in the frequency range of 1 to 20 Hz that is representative of anthropogenic noise sources (human activities, traffic and machineries). The lockdown effects led to a 1–19 dB decrease in seismic noise levels (maximum change in PSD values) in the 5–15 Hz at different stations, based on their location. Further, a drastic fall in noise level (27–79%) is observed during the daytime in urban areas, in the 5–15 Hz frequency range. However, no major changes are observed in diurnal or hourly patterns at stations installed in remote locations. The effect of subsurface geology is also evident in the seismic noise records. Due to the restrictions during the lockdown, the effect of cultural noise is effectively removed from the background seismic noise. Thus, the noise level in the 1–20 Hz observed during the lockdown period is representative of other sources, mainly the natural response of the subsurface geology (e.g., amplification due to site response) and/or other environmental factors. Most of the earlier studies related to subsurface geology were restricted to 1–10 Hz. In the present study, the effect of higher predominant frequencies (10–20 Hz), contributing to large seismic amplitudes is clearly witnessed in hard rock formations. However, no changes in the noise levels are observed for frequencies below 1 Hz, where the contribution of cultural noise is small and microseism noise sources dominate. The present study provides an overview on seismic background noise levels from the perspective of anthropogenic sources and subsurface geology, which would be very helpful in selecting sites for installation of seismic stations in the future.

## Supplementary Information

Supplementary Information 1.

Supplementary Information 2.
