# Supplementary material for: Effect of coronavirus lockdowns on the ambient seismic noise levels in Gujarat, northwest India
Source: Sci Rep. 2021 Mar 30;11:7148. doi: 10.1038/s41598-021-86557-9 (PMC8010099; doi:10.1038/s41598-021-86557-9)
Supplement: Supplementary file 1 — Supplementary Information 1. [file 41598_2021_86557_MOESM1_ESM.pdf]

Supplementary Information for

Effect of Coronavirus lockdowns on the ambient seismic noise levels in Gujarat, northwest India

Ketan Singha Roy[1], Jyoti Sharma[1,*], Santosh Kumar[1], and M. Ravi Kumar[2]

[1]Institute of Seismological Research, Gandhinagar, 382009, India.

[2] National Geophysical Research Institute, Hyderabad, 500007, India.

Contents of this file

Supplementary Figures S1 to S12

Figures

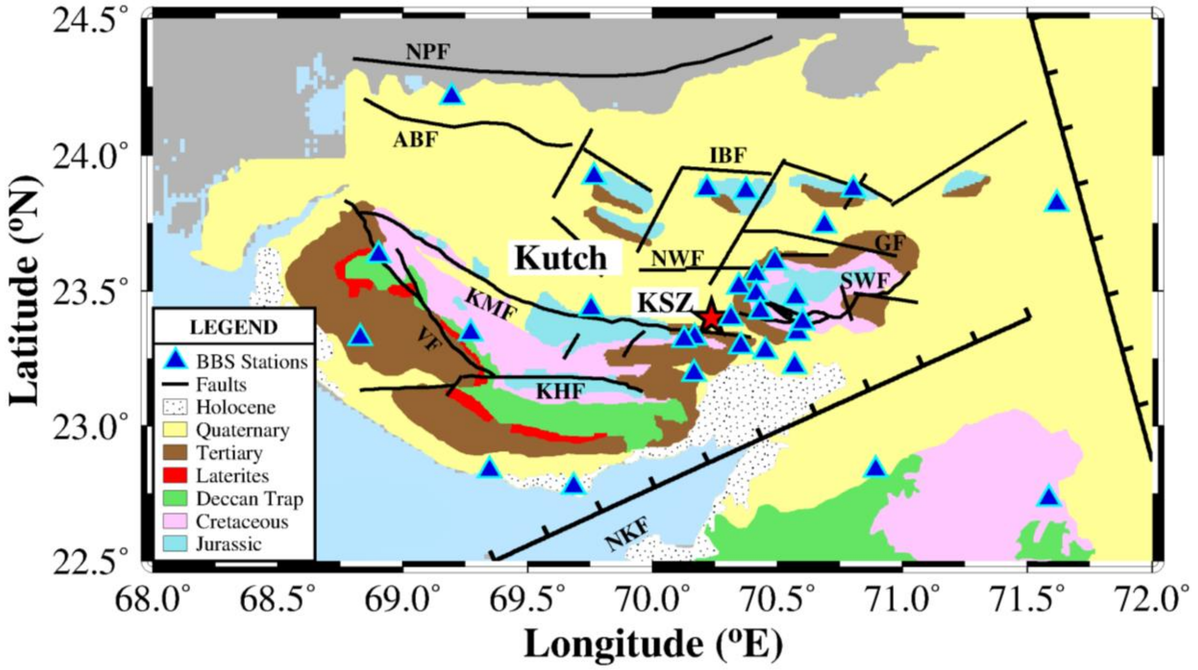

Figure S1: Distribution of the BBS stations of ISR seismic network in the Kutch region of Gujarat. The geological and tectonic features are also marked [12-14]. The major faults in the study region are Nagar Parkar Fault (NPF), Allah Bund Fault (ABF), Island Belt Fault (IBF), Gedi Fault (GF), North Wagad Fault (NWF), South Wagad Fault (SWF), Kutch Mainland Fault (KMF), Katrol Hill fault (KHF), Vigodi Fault (VF) and North Kathiawar Fault (NKF). Epicenter of the 2001 Bhuj earthquake is represented with red coloured star. Kutch Seismic Zone (KSZ) is also marked. This fig. is prepared using PyGMT (v0.3.0; https://www.pygmt.org/v0.3.0/) [24].

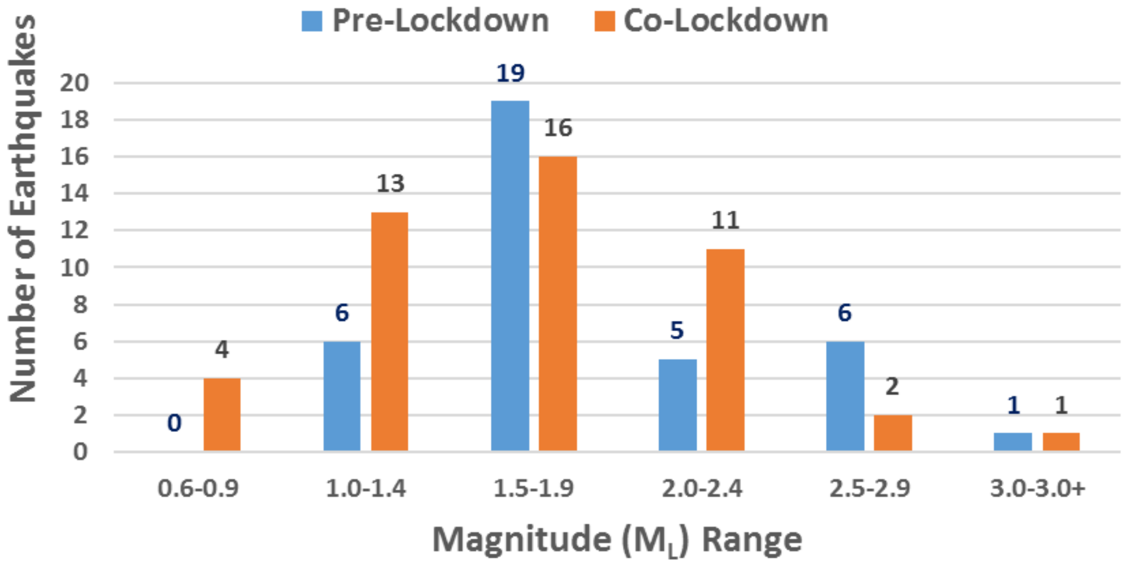

Figure S2: Number of earthquakes recorded in the seismically active zone of Kutch region (KSZ) during Pre-lockdown (01/03/2020–24/03/2020) and Co-lockdown (25/03/2020–20/04/2020). Matplotlib is used during preparation of figs. S2-S12 using Python in Jupyter notebook [25-27].

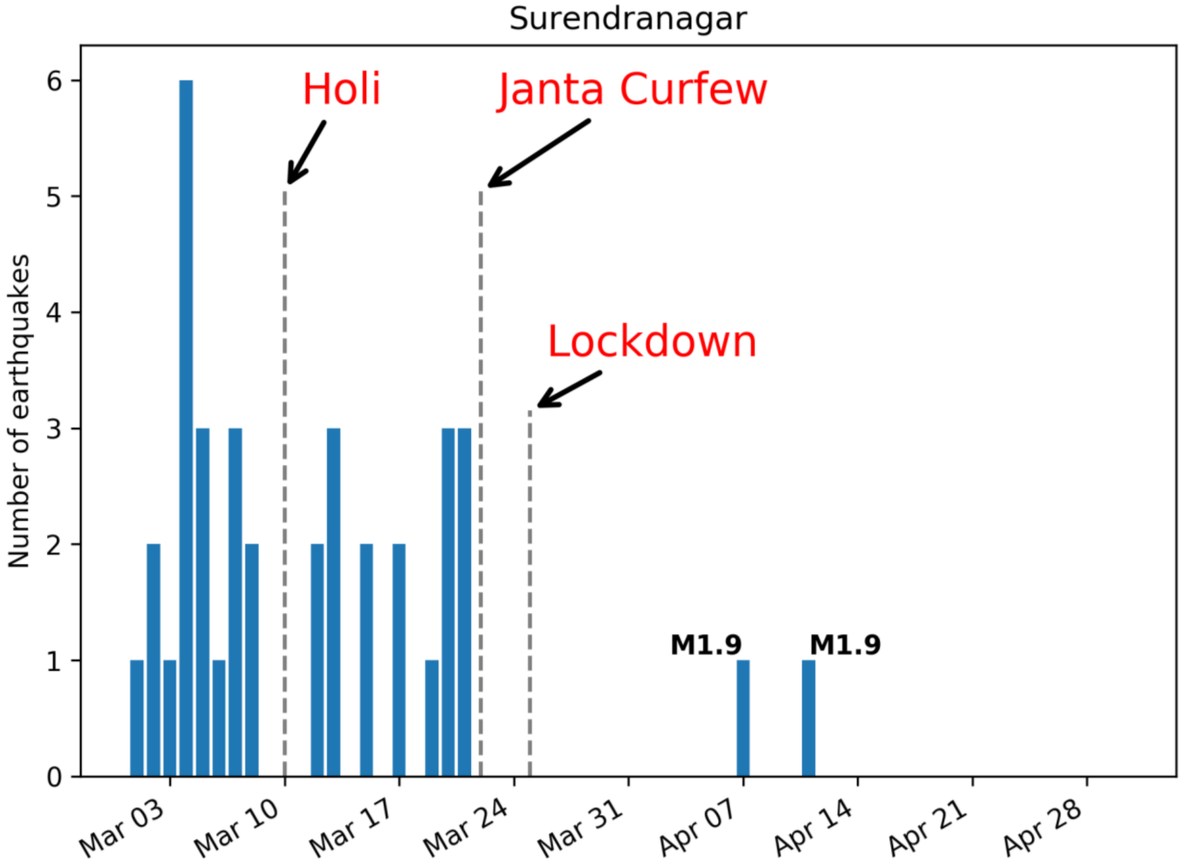

Figure S3: Seismic records of earthquakes and blasts recorded in the Surendranagar region of Saurashtra, Gujarat. However, during lockdown only two events (M 1.9) were recorded. Also, no seismic records on Public Holiday (Holi) and Janta Curfew affirms ISR's claims of quarry blast activity.

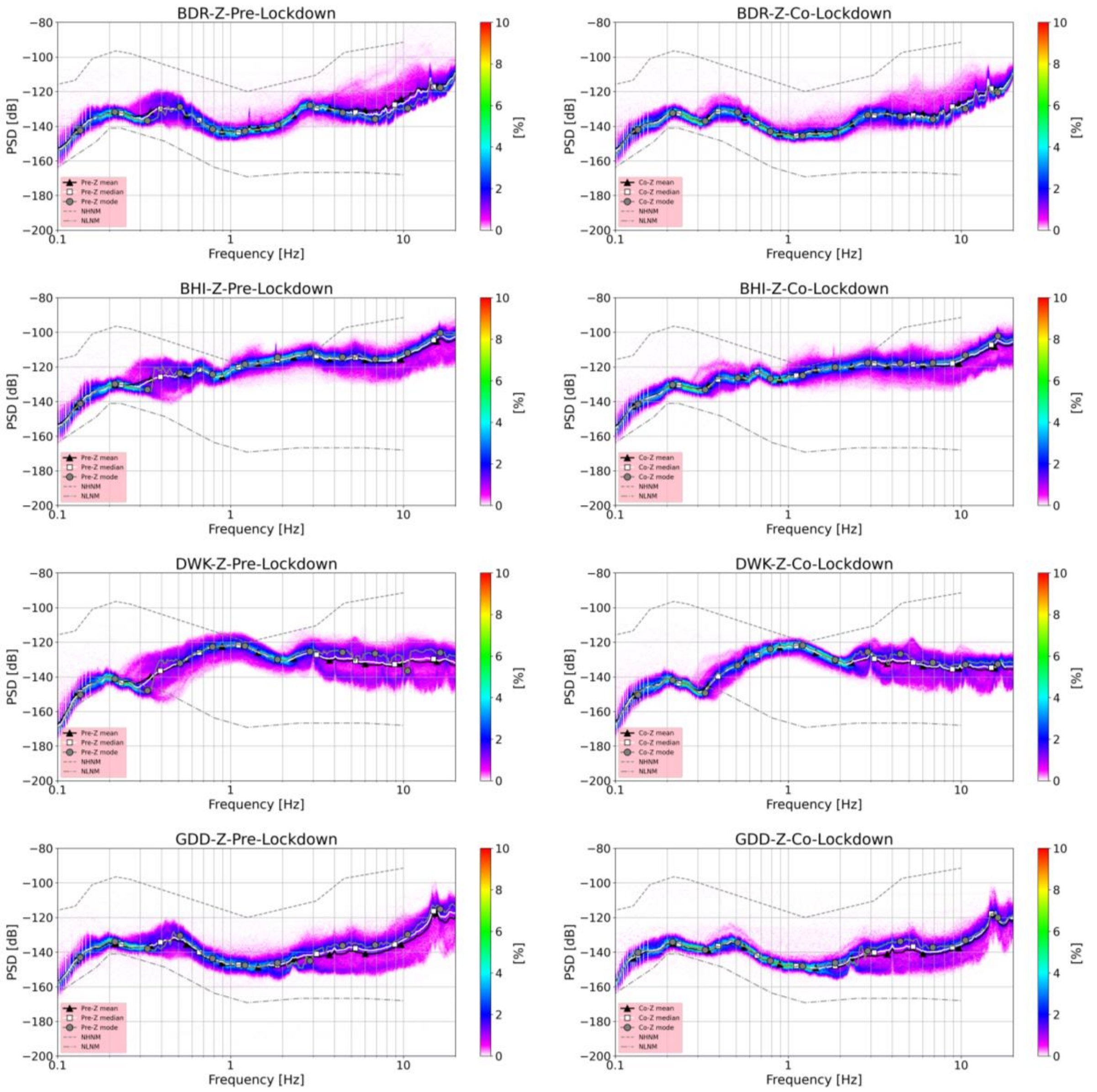

Figure S4: PDF distribution computed using PSD for the vertical (Z) components of different stations of ISR seismic network, shown for the pre- and co-lockdown periods. The variation in seismic noise levels in terms of mean, median and mode is also shown. The new high and new low noise models (NHNM, NLNM) [9] are also represented by gray lines, as upper and lower limits.

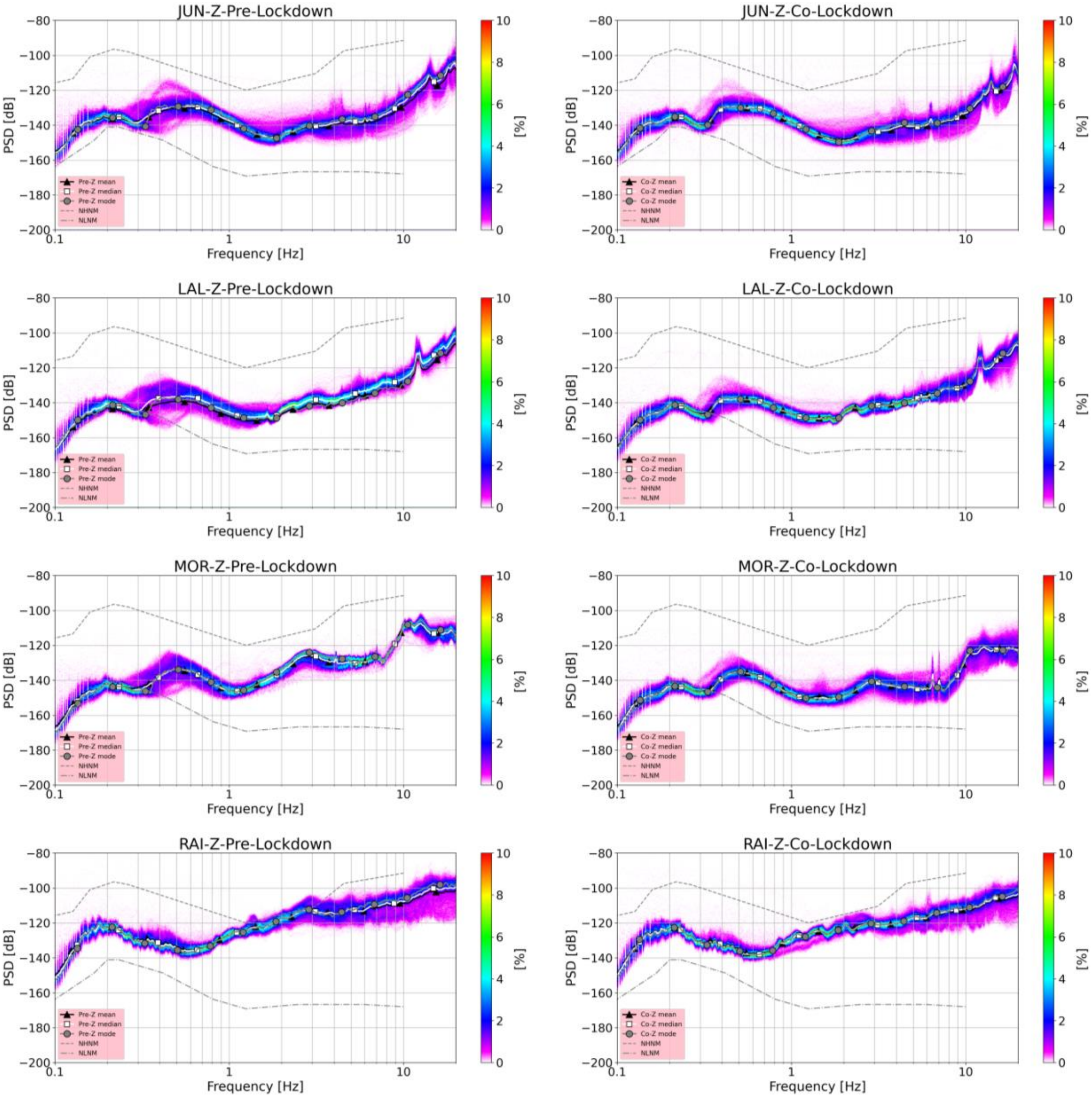

Figure S4: (Contd.)

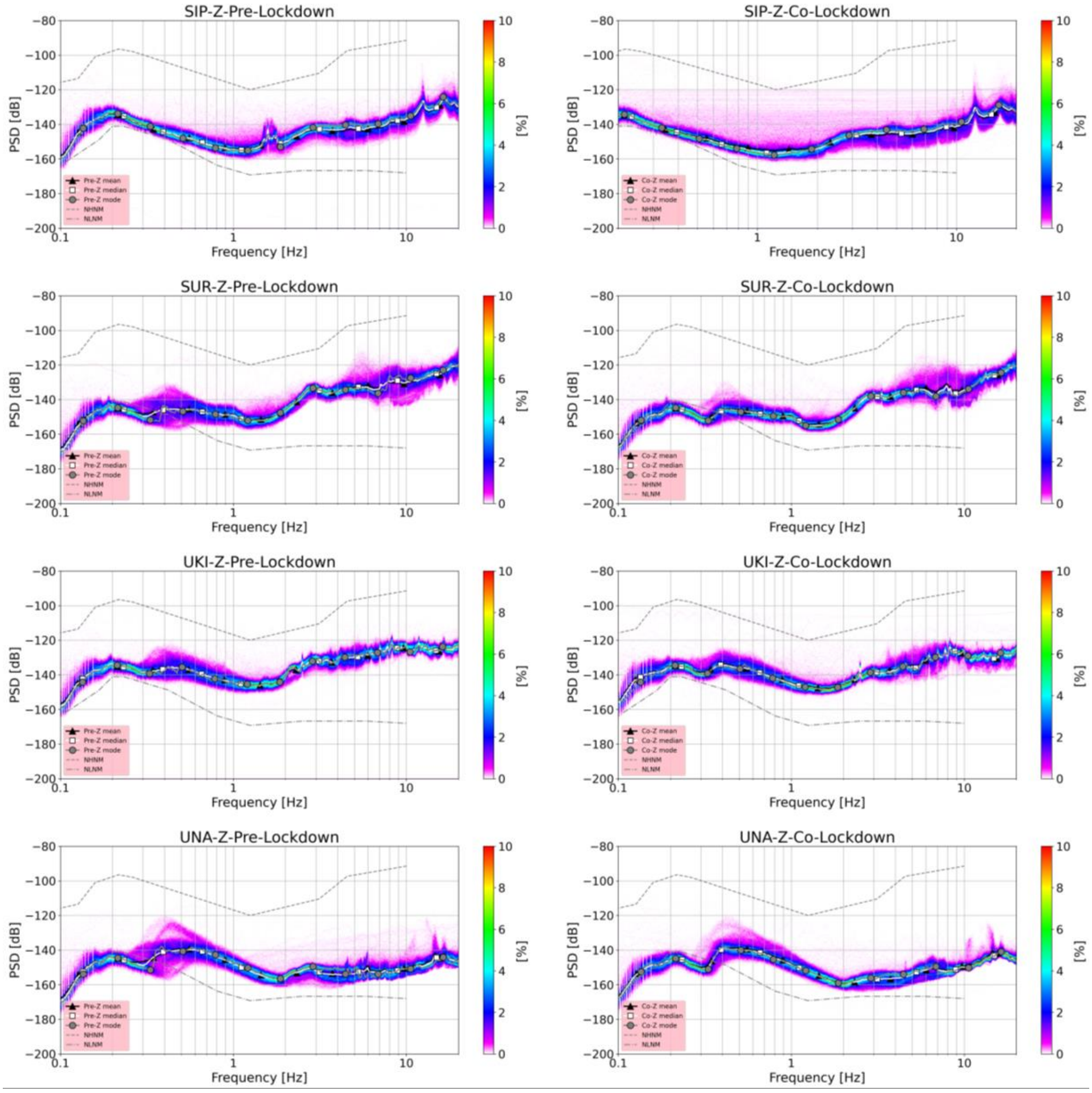

Figure S4: (Contd.)

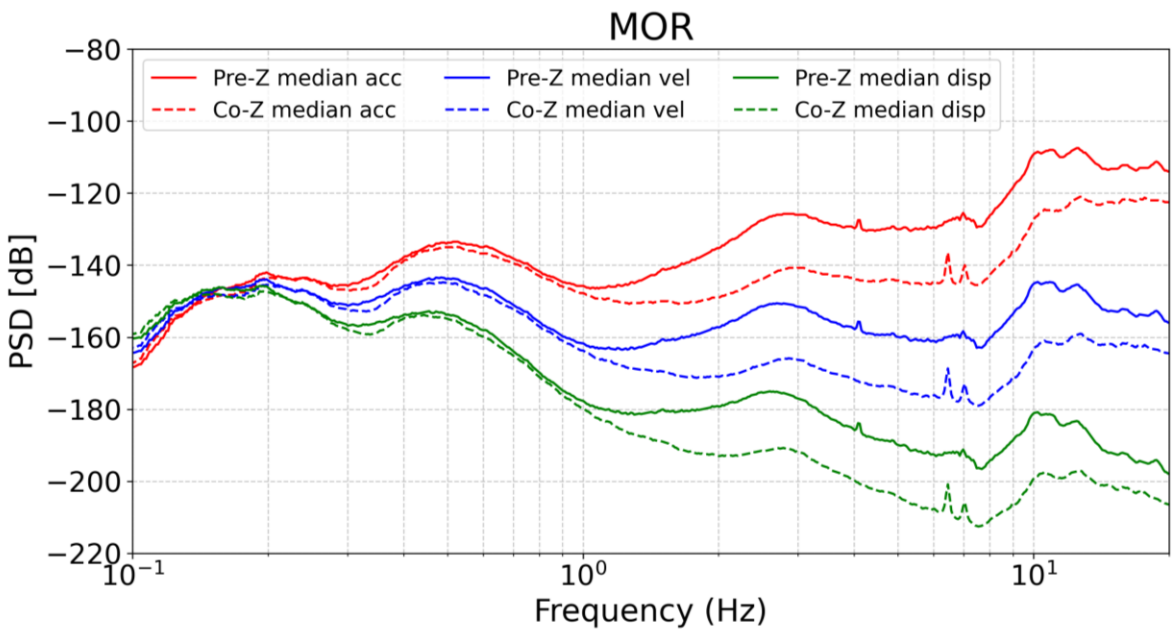

Figure S5: Variation in median PSD estimates with respect to acceleration, velocity and displacement for pre- and co-lockdown durations for station MOR. Following equations are used to estimate the PSD with respect to acceleration (P_a[dB]), velocity (P_v[dB]) and displacement (P_d[dB]) in frequency domain [10]. The power spectral density P_a of acceleration relative to the metric unit $1*(m/s^2)^2/Hz$, we get $P_a[dB]=10\log_{10}[P_a/1*(m/s^2)^2/Hz]$, $P_v[dB]=P_a[dB]+20\log_{10}(T/2\pi)$ and $P_d[dB]=P_a[dB]+40\log_{10}(T/2\pi)$ or $P_d[dB]=P_v[dB]+20\log_{10}(T/2\pi)$. Here, T represents the period.

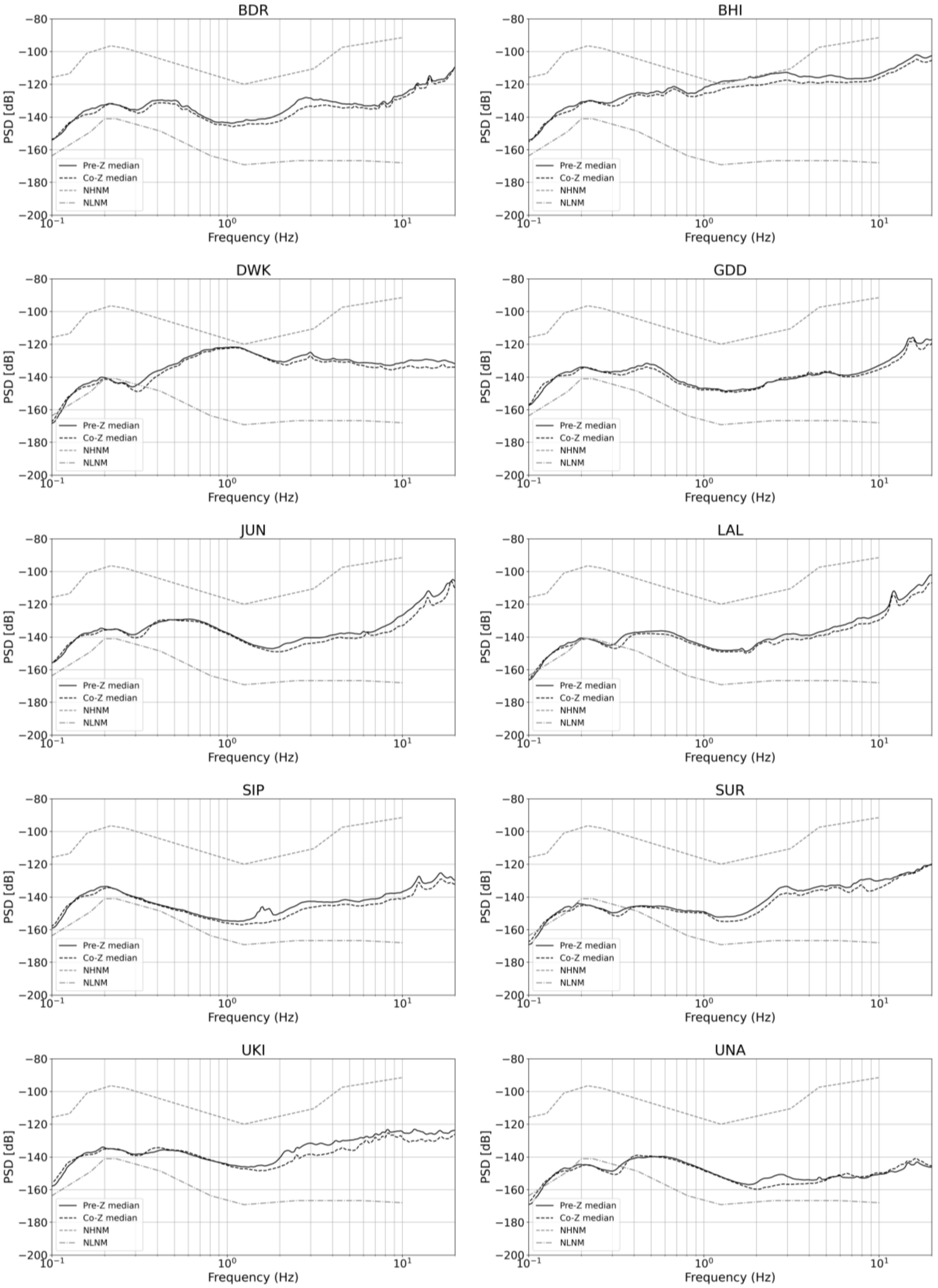

Figure S6: Variation in seismic background noise level in terms of PSD, with respect to frequency, using median for the vertical (Z) components of different stations of ISR seismic network for pre- and co-lockdown periods. The new high and new low noise models (NHNM, NLNM) [9] are also represented by gray lines, as upper and lower limits.

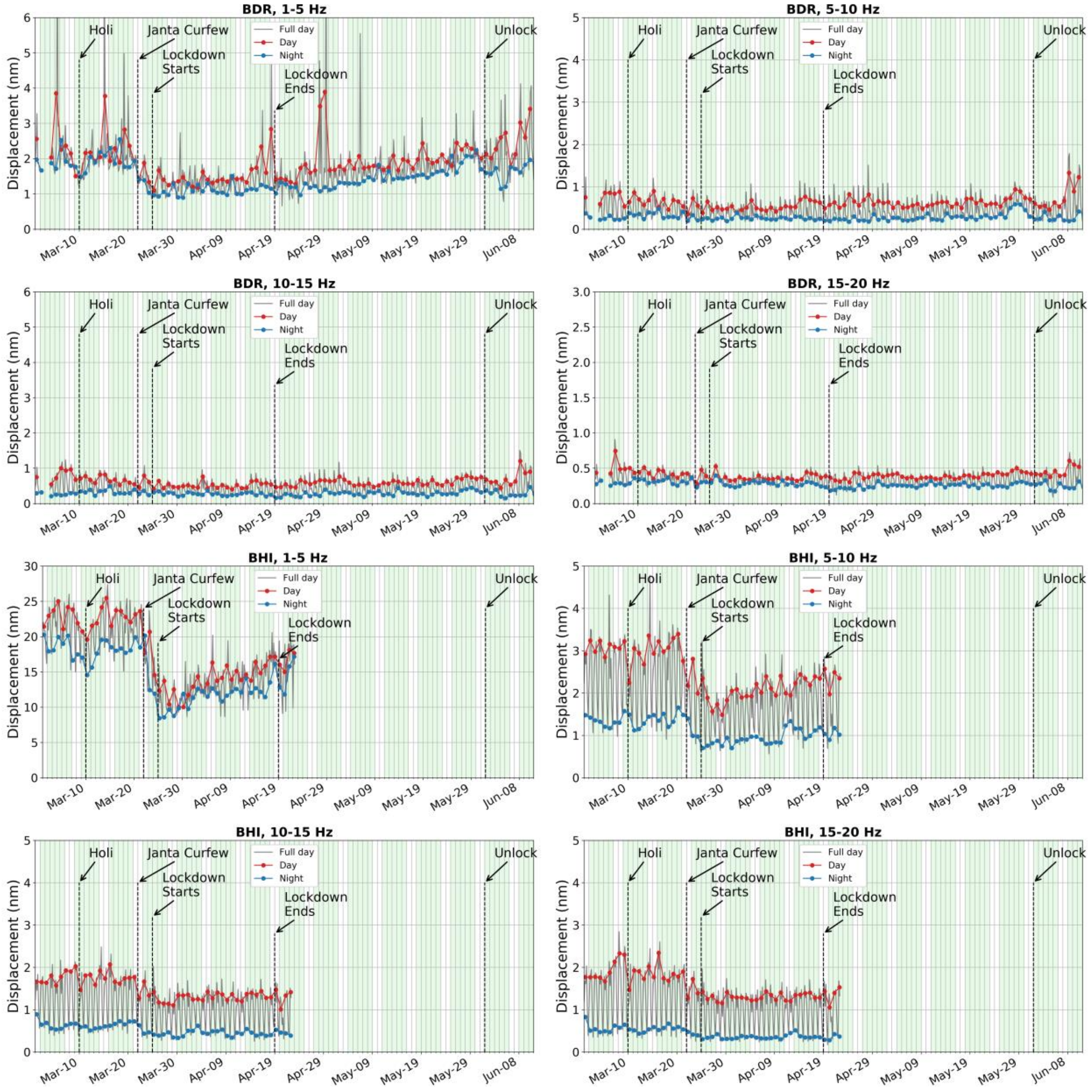

Figure S7: Day wise ground displacement variation in terms of median d_{rms} for the pre-, co- and post-lockdown periods at different stations of ISR seismic network, in different high frequency ranges (1-5 Hz, 5-10 Hz, 10-15 Hz and 15-20 Hz) between 1-20 Hz. The variation in ground displacement during daytime and nighttime is also shown. The complete lockdown period, beginning of Unlock, Janta Curfew and extended holiday during Holi (Indian Festival) are also marked.

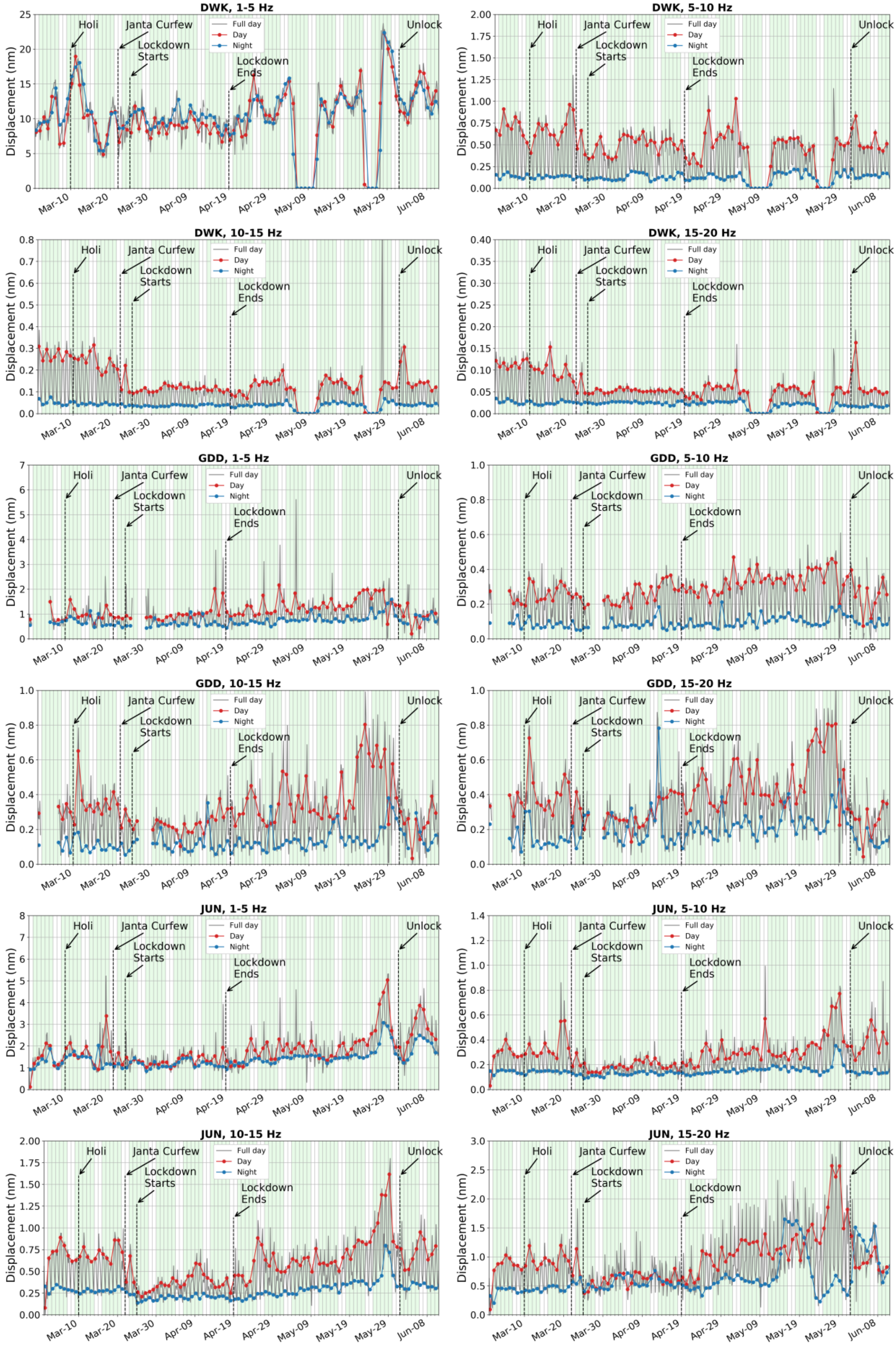

Figure S7: (Contd.)

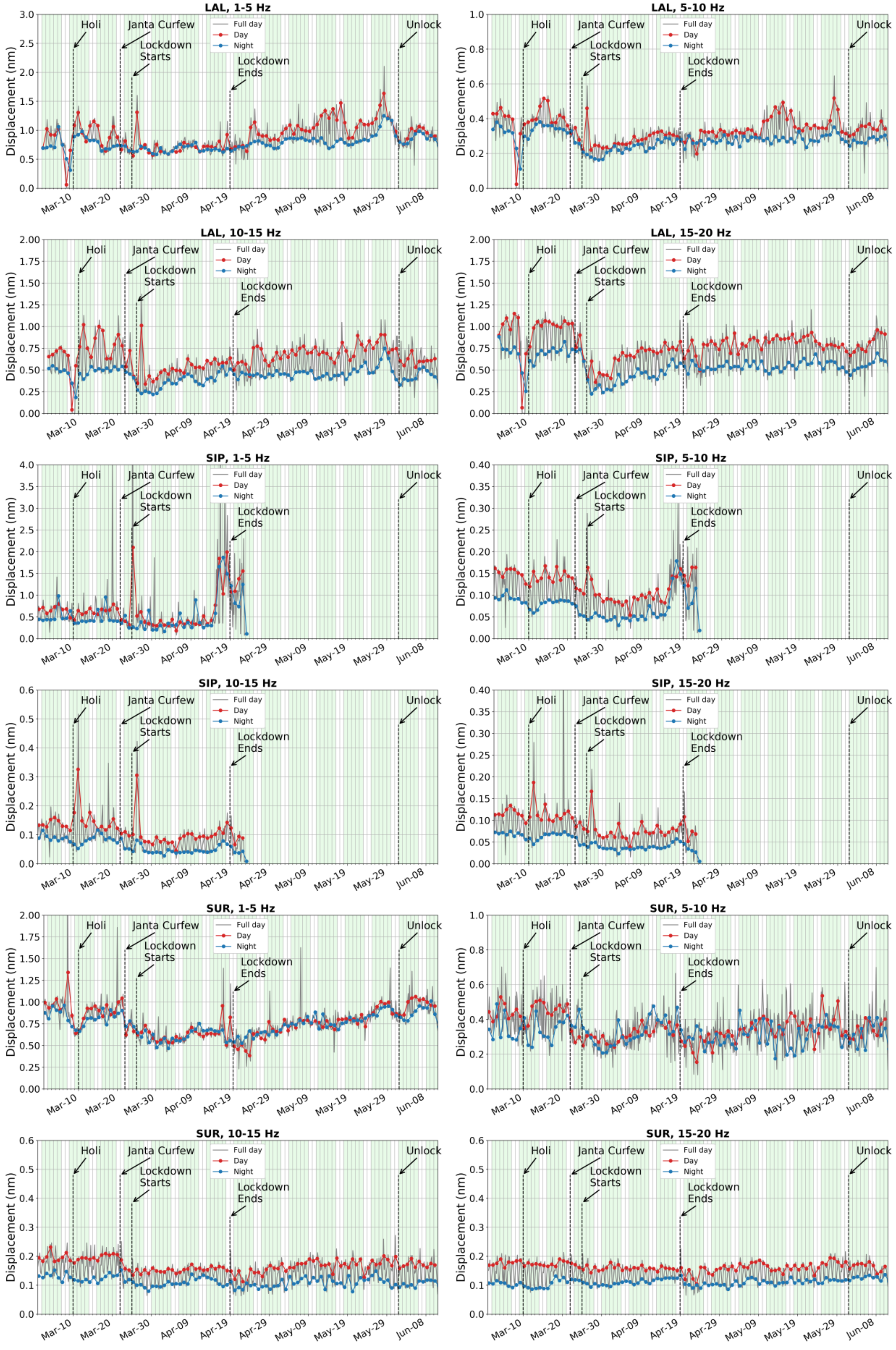

Figure S7: (Contd.)

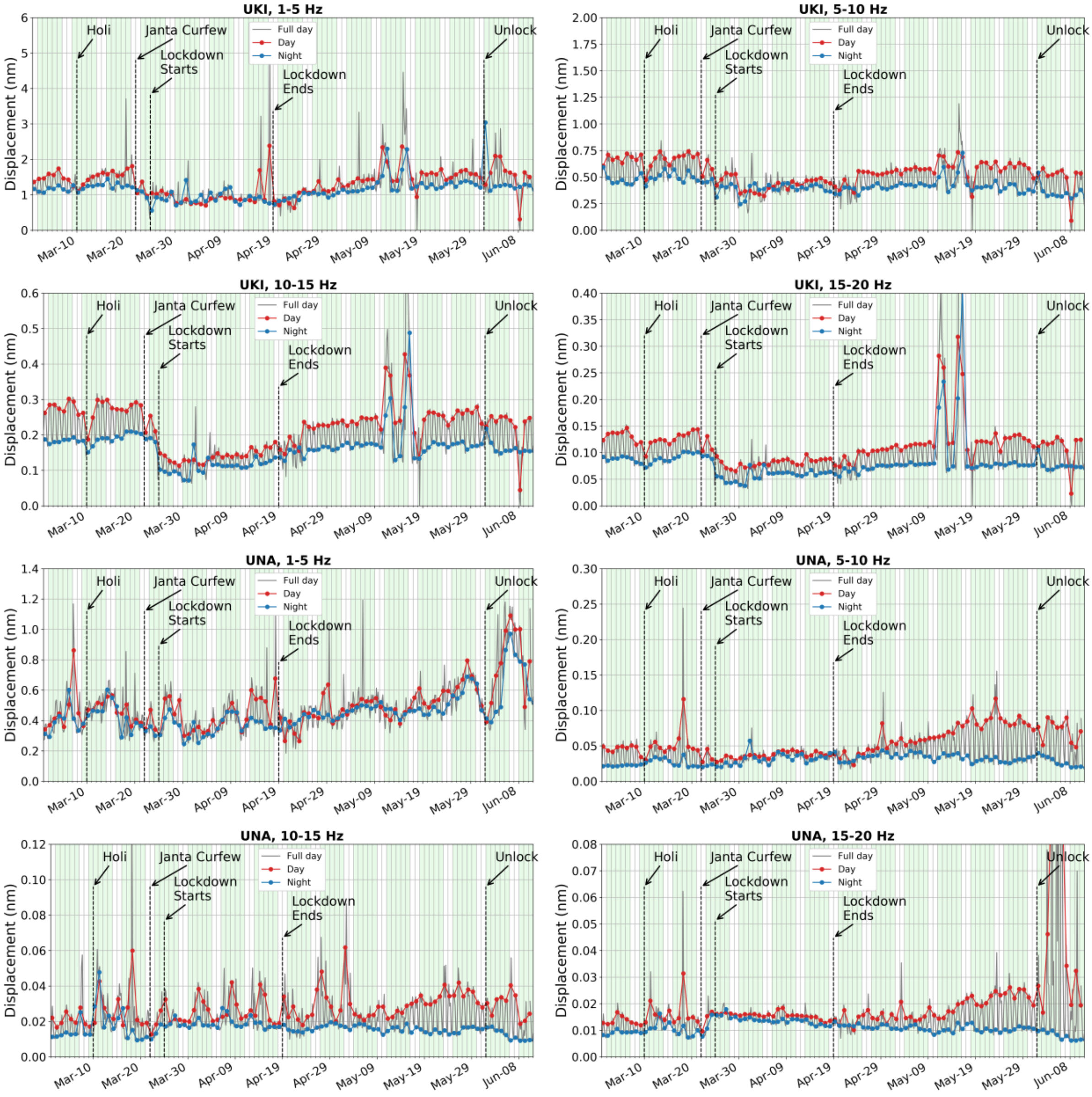

Figure S7: (Contd.)

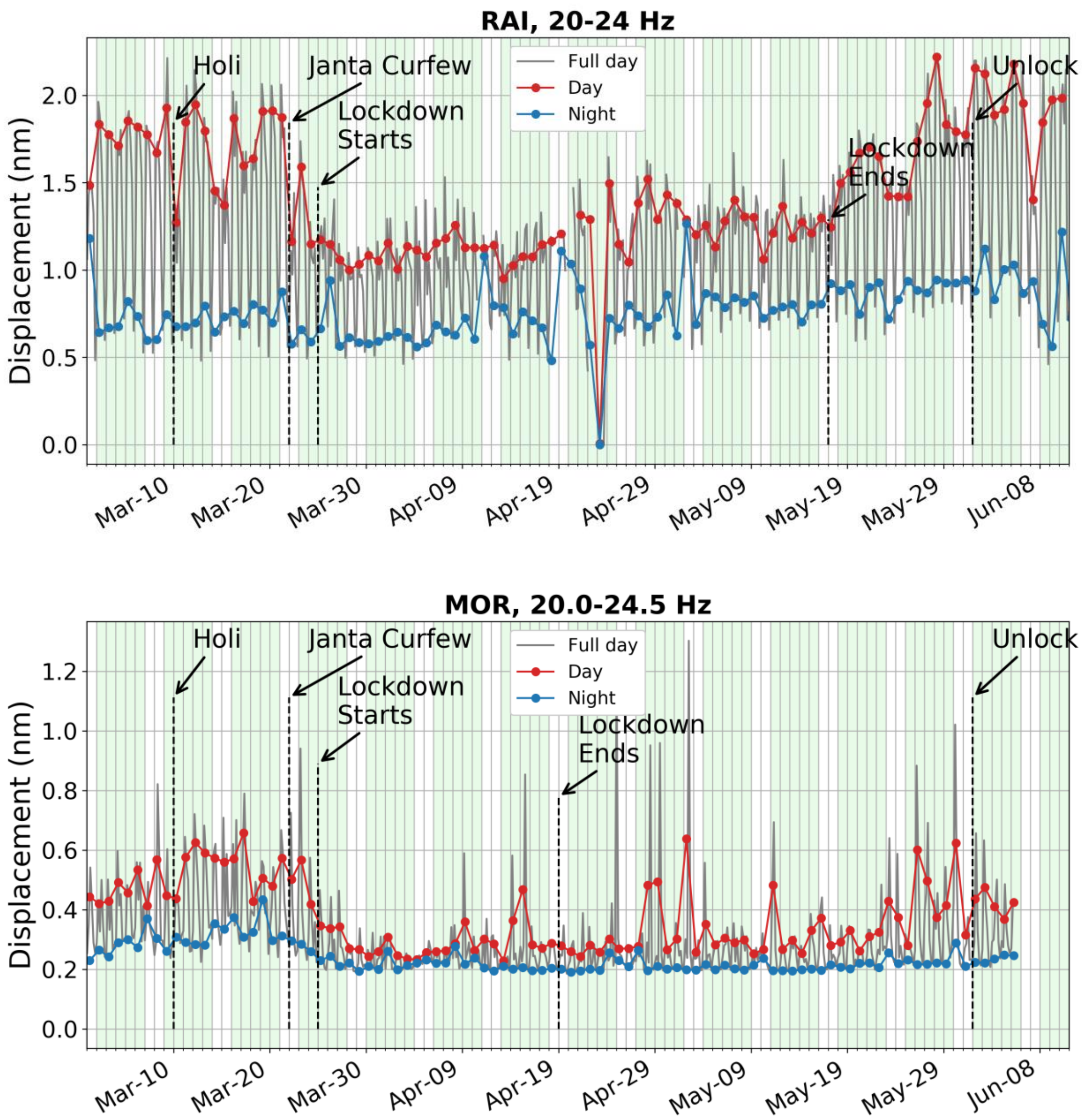

Figure S8: Day wise ground displacement variation in terms of median d_{rms} for the pre-, co- and post-lockdown periods at stations RAI and MOR of ISR seismic network in the frequency range of 20 to ~25 Hz. Different higher limits (RAI 24 Hz, MOR 24.5 Hz) are used in order to eliminate the effect of nyquist frequency. The variation in ground displacement during daytime and nighttime is also shown. The complete lockdown period, beginning of Unlock, Janta Curfew and extended holiday during Holi (Indian Festival) are also marked.

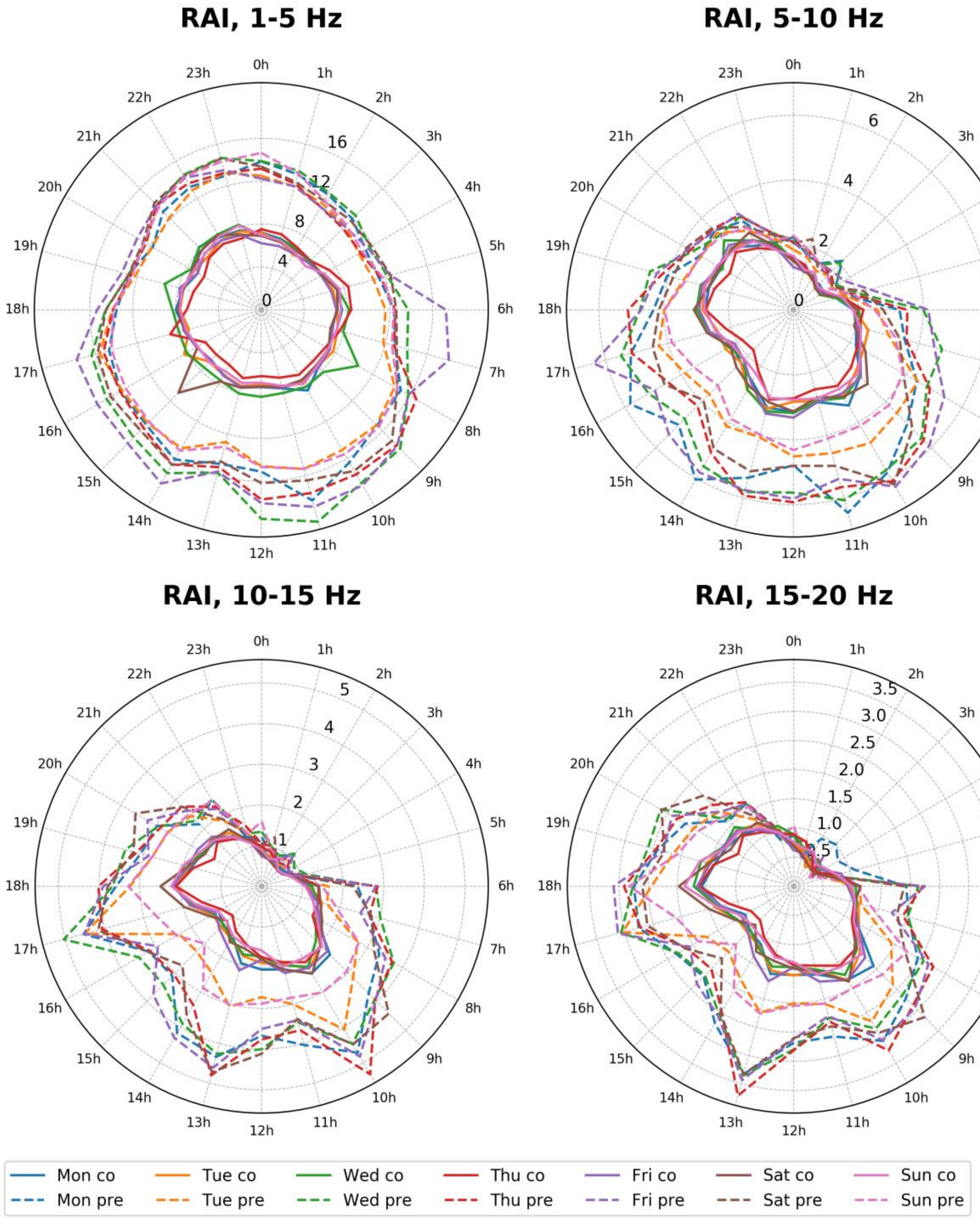

Figure S9: Hour wise variation in seismic background noise in terms of ground displacement (d_{rms}, in nm) for pre- and co-lockdown periods at RAI station for different weekdays and frequency ranges between 1 and 20 Hz. The d_{rms} value on any particular day is representative of the median value of all the corresponding days for pre- and co-lockdown periods. Here, time is mentioned in Indian Standard time (IST).

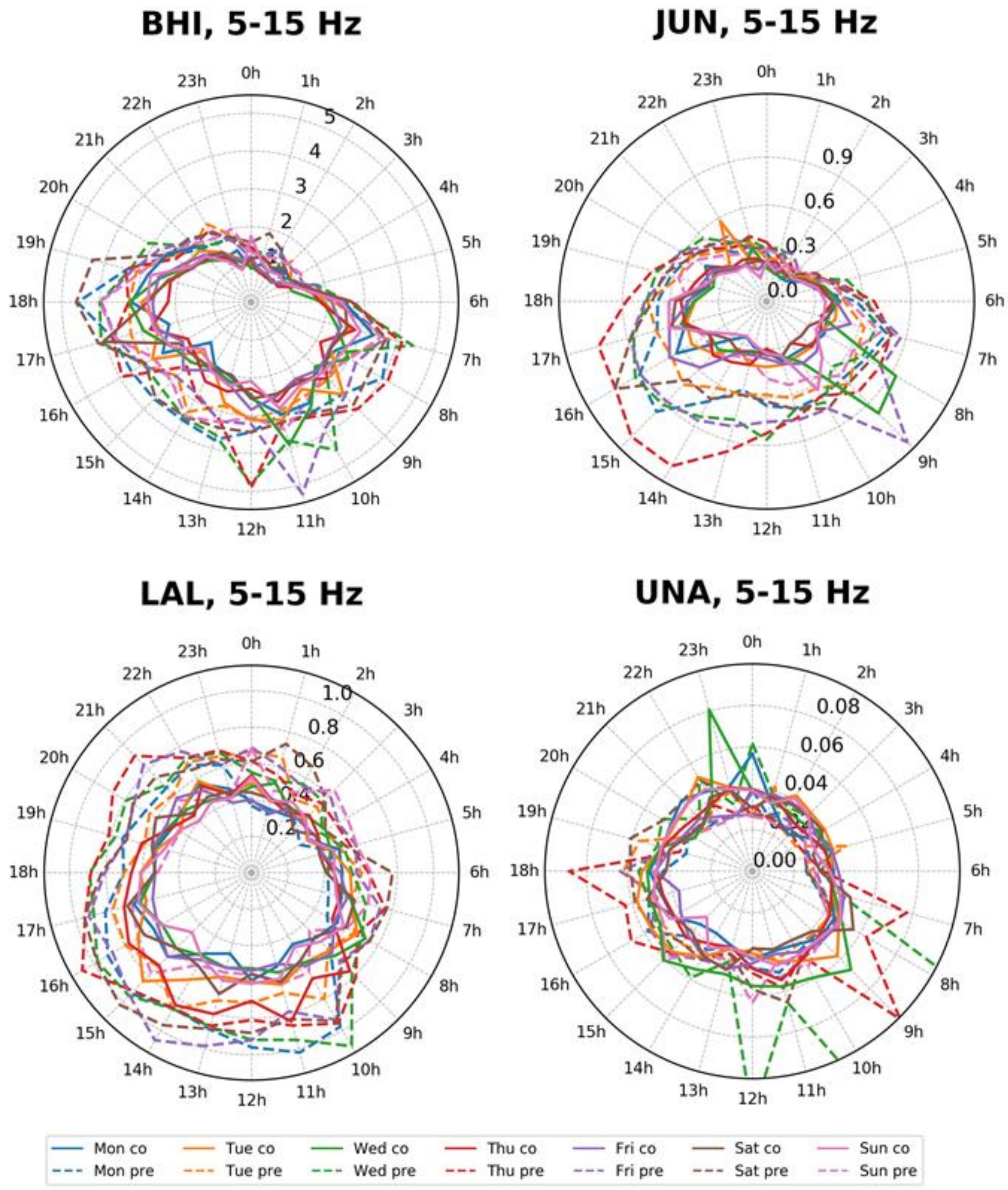

Figure S10: Hour wise variation in seismic background noise in terms of ground displacement (d_{rms}, in nm) for pre- and co-lockdown periods at different stations for different weekdays, in the frequency range of 5 to 15 Hz. The d_{rms} value on any particular day is representative of the median value of all the corresponding days for pre- and co-lockdown periods. Here, time is mentioned in Indian Standard time (IST). Subsurface geology and other local anthropogenic conditions are mentioned in Table 1.

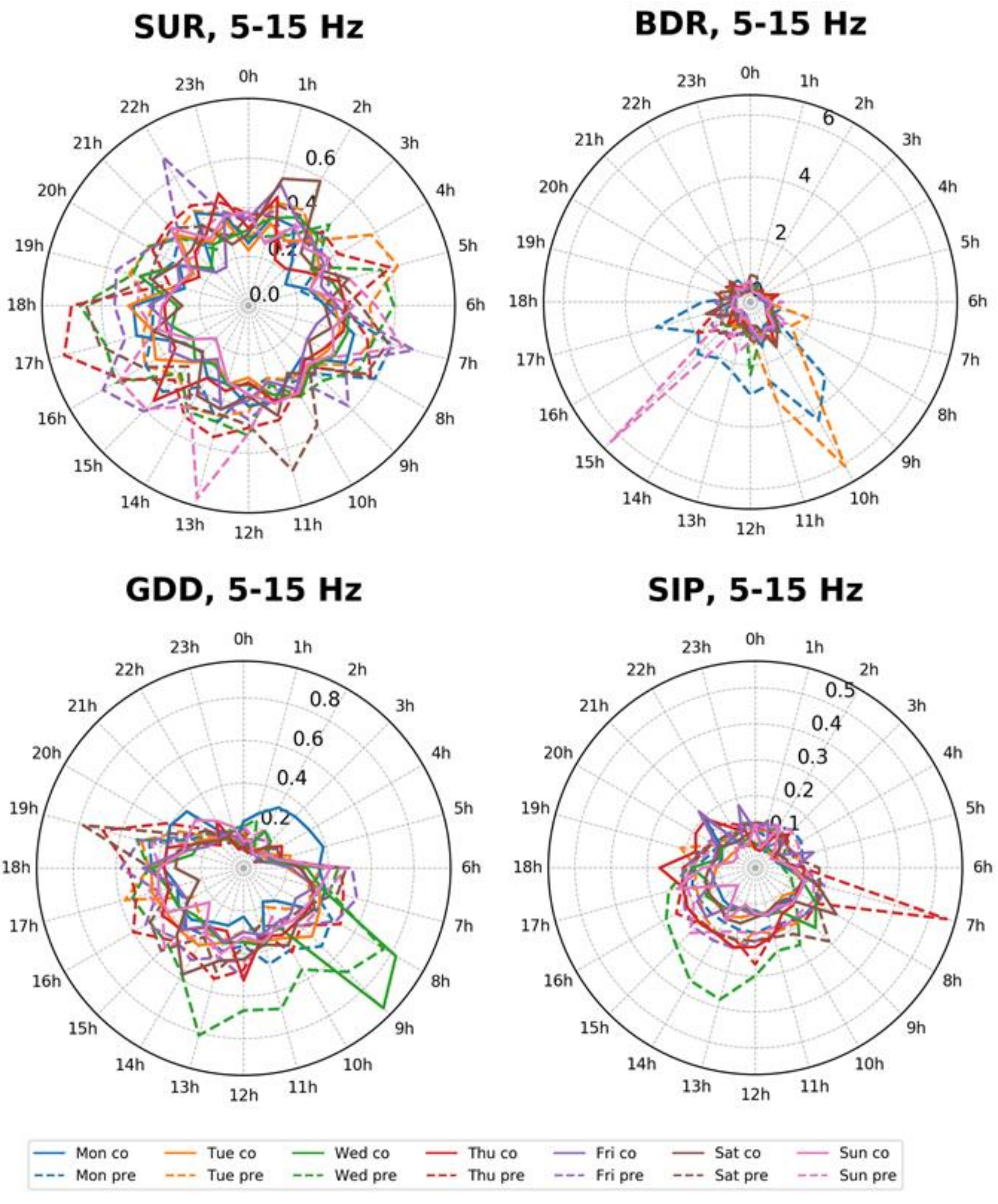

Figure S10: (Contd.)

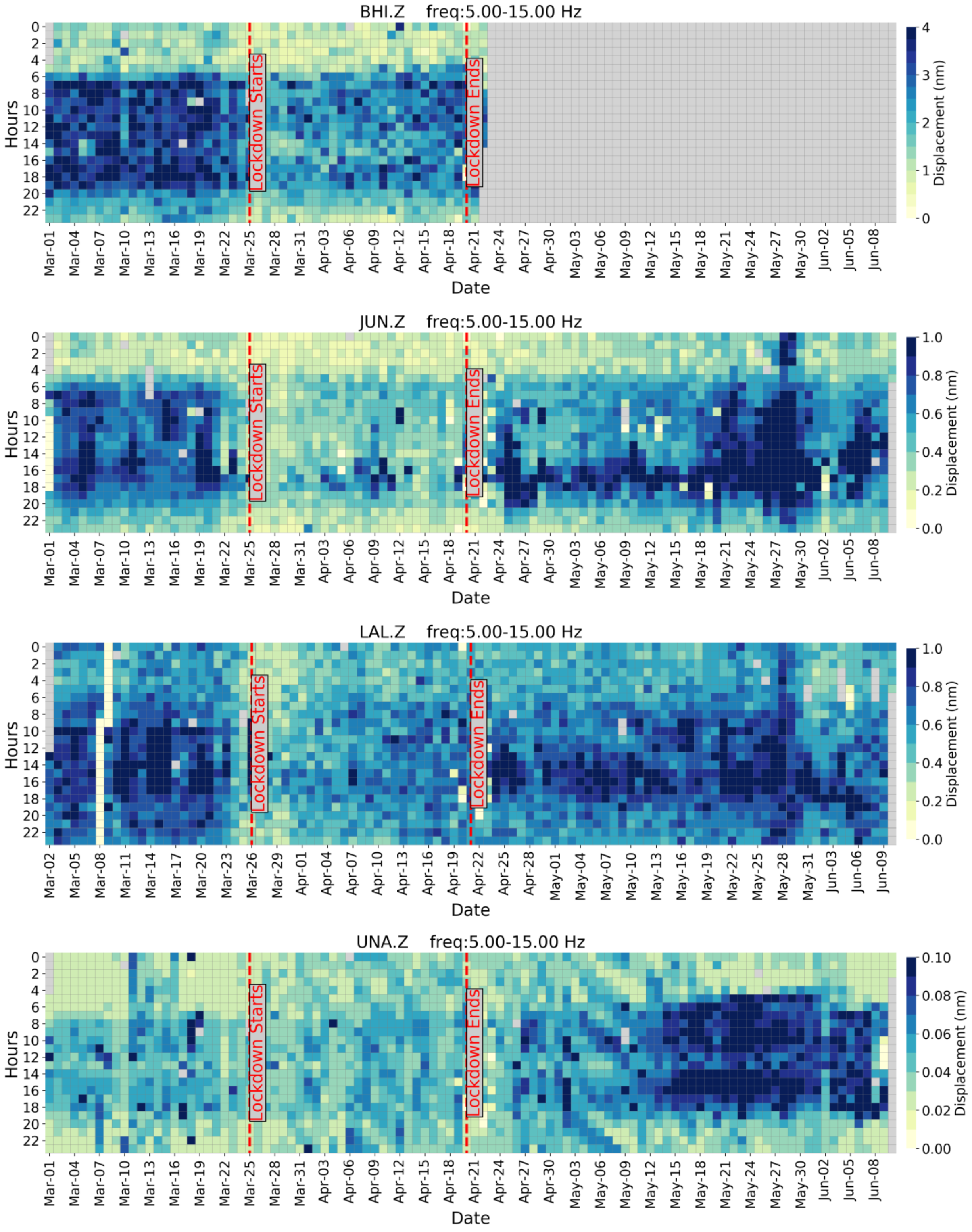

Figure S11: Day wise hourly variation in ground displacement (d_{rms}, in nm) during pre-, co- and post-lockdown periods at different stations in the frequency range of 5-15 Hz. Here, data are normalized to the 15-85th percentiles of the pre-lockdown period for better representation and comparison purpose. Gray colour represents data gap. Time is mentioned in IST. Subsurface geology and other local anthropogenic conditions are mentioned in Table 1.

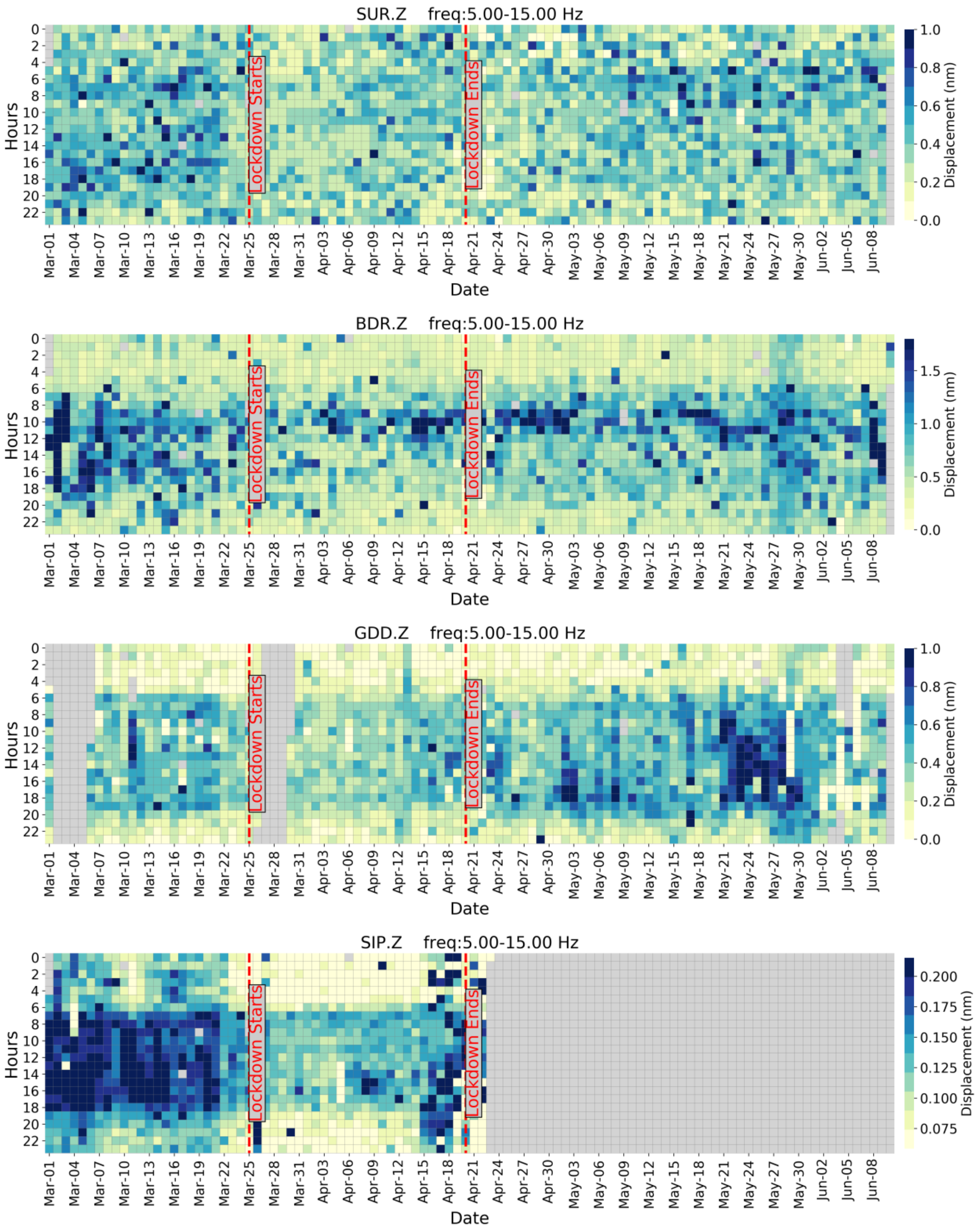

Figure S11: (Contd.)

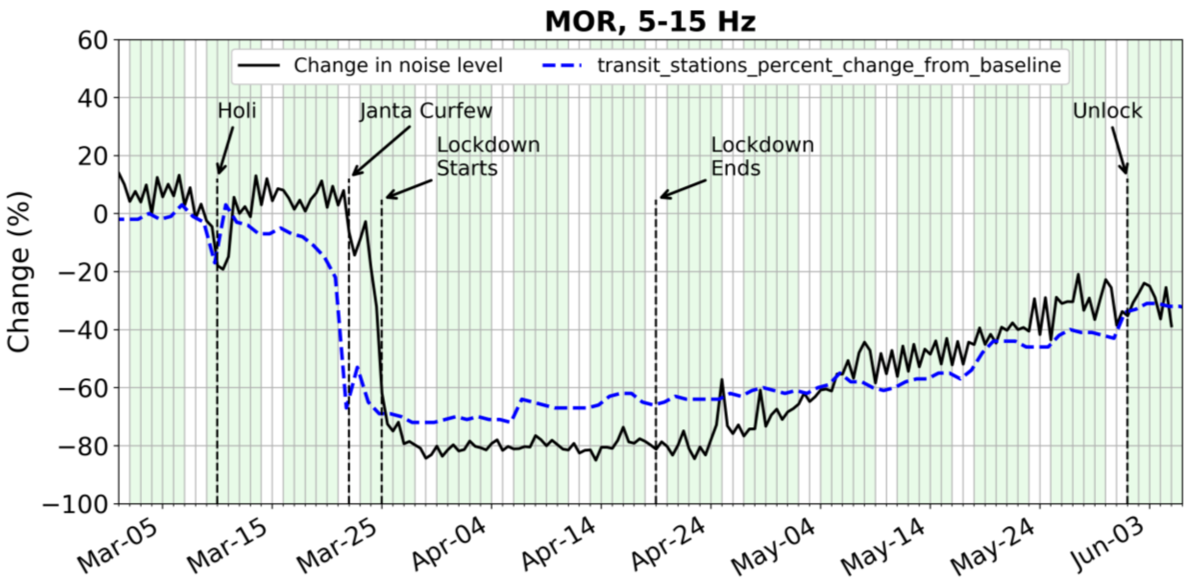

Figure S12: Day wise change in seismic noise level in median ground displacement (d_{rms}) during pre-, co- and post-lockdown periods at station MOR, in the 5-15 Hz frequency range. To estimate the change in the seismic noise level in the d_{rms} values for the pre-, co- and post-lockdown periods, the median value obtained for the pre-lockdown period is considered as the baseline. Mobility data (transport) for the Gujarat region is also plotted for the same duration for comparison and to show a one-to-one correspondence between ambient noise sources and seismic noise level records. Mobility data for the Gujarat, India is available at https://www.google.com/covid19/mobility/.
