# Supplementary material for: Effect of coronavirus lockdowns on the ambient seismic noise levels in Gujarat, northwest India
Source: Sci Rep. 2021 Mar 30;11:7148. doi: 10.1038/s41598-021-86557-9 (PMC8010099; doi:10.1038/s41598-021-86557-9)
Supplement: Supplementary file 2 — Supplementary Information 2. [file 41598_2021_86557_MOESM2_ESM.pdf]

Supplementary Information for

Effect of Coronavirus lockdowns on the ambient seismic noise levels in Gujarat, northwest India

Ketan Singha Roy[1], Jyoti Sharma[1,*], Santosh Kumar[1], and M. Ravi Kumar[2]

[1]Institute of Seismological Research, Gandhinagar, 382009, India.

[2] National Geophysical Research Institute, Hyderabad, 500007, India.

Contents of this file

Supplementary Table S1-S3

Table S1: Range of the estimated population mean of seismic noise levels in PSD (dB) with 95% confidence level in different frequency ranges for Pre- and Co-lockdown periods. The range is also estimated for the population mean observed in difference in PSD estimates between Pre- and Co-lockdown periods.

**Stations**	**Geology**	**Duration/ Change**	**Frequency Range**	
			**5 - 15 Hz**	**15 - 20 Hz**
BHI	Quaternary Sediments	Pre-lockdown	(-115.0, -113.9)	(-121.7, -120.2)
		Co-lockdown	(-117.5, -116.3)	(-117.5, -116.3)
		Pre-Co Difference	(1.7, 3.3)	(1.6, 3.6)
RAI	Quaternary Sediments	Pre-lockdown	(-109.5, -108.4)	(-121.2, -119.5)
		Co-lockdown	(-113.7, -112.6)	(-124.2, -122.8)
		Pre-Co Difference	(3.4, 5.0)	(2.0, 4.2)
DWK	Tertiary Sediments	Pre-lockdown	(-132.4, -132.1)	(-133.7, -132.4)
		Co-lockdown	(-135.0, -134.6)	(-135.4, -134.1)
		Pre-Co Difference	(2.3, 2.9)	(0.8, 2.6)
MOR	Deccan Traps	Pre-lockdown	(-121.2, -118.2)	(-134.7, -132.8)
		Co-lockdown	(-136.5, -133.3)	(-142.1, -140.7)
		Pre-Co Difference	(13.0, 17.5)	(6.5, 8.9)
JUN	Deccan Traps	Pre-lockdown	(-132.1, -129.2)	(-136.3, -134.9)
		Co-lockdown	(-135.1, -132.5)	(-137.7, -136.4)
		Pre-Co Difference	(1.2, 5.0)	(0.4, 2.4)
LAL	Deccan Traps	Pre-lockdown	(-130.3, -128.0)	(-140.6, -138.9)
		Co-lockdown	(-130.2, -127.8)	(-139.8, -138.2)
		Pre-Co Difference	(-1.8, 1.5)	(-2.0, 0.4)
UKI	Deccan Traps	Pre-lockdown	(-126.5, -125.8)	(-135.8, -134.7)
		Co-lockdown	(-130.3, -129.5)	(-137.7, -136.7)
		Pre-Co Difference	(3.3, 4.3)	(1.2, 2.7)
UNA	Deccan Traps	Pre-lockdown	(-150.5, -149.6)	(-149.2, -148.3)
		Co-lockdown	(-149.8, -148.7)	(-149.9, -148.9)
		Pre-Co Difference	(-1.5, -0.12)	(0.04, 1.4)
SUR	Cretaceous Rocks	Pre-lockdown	(-130.6, -129.8)	(-141.8, -140.3)
		Co-lockdown	(-132.9, -131.9)	(-143.5, -142.0)
		Pre-Co Difference	(1.6, 2.9)	(0.7, 2.8)
BDR	Jurassic Rocks	Pre-lockdown	(-126.5, -124.6)	(-132.9, -131.6)
		Co-lockdown	(-128.9, -127.0)	(-135.1, -133.8)
		Pre-Co Difference	(1.1, 3.7)	(1.3, 3.1)
GDD	Jurassic Rocks	Pre-lockdown	(-136.5, -134.5)	(-139.9, -138.7)
		Co-lockdown	(-137.1, -135.4)	(-140.3, -139.2)
		Pre-Co Difference	(-0.6, 2.1)	(-0.4, 1.2)
SIP	Proterozoic Rocks	Pre-lockdown	(-138.8, -137.2)	(-143.6, -142.5)
		Co-lockdown	(-142.4, -140.9)	(-145.6, -144.5)
		Pre-Co Difference	(2.6, 4.8)	(1.2, 2.8)

Table S2: Range of the estimated population mean of seismic noise levels in d_{rms} (in nm) for daytime, with 95% confidence level in different frequency ranges for Pre- and Co-lockdown periods. The range is also estimated for the population mean observed in the difference in d_{rms} estimates between Pre- and Co-lockdown periods.

Stns.	Geology	Duration	Frequency Range				
			1 - 5 Hz	5 - 10 Hz	10 - 15 Hz	5 - 15 Hz	15 - 20 Hz
BHI	Quaternary Sediments	Pre-lockdown	21.71 - 22.29	2.86 - 3.00	1.69 - 1.76	3.35 - 3.50	1.80 - 1.87
		Co-lockdown	13.62 - 14.15	2.02 - 2.12	1.27 - 1.32	2.41 - 2.52	1.28 - 1.33
		Pre-Co Difference	7.72 - 8.51	0.77 - 0.95	0.39 - 0.47	0.87 - 1.05	0.50 - 0.58
RAI	Quaternary Sediments	Pre-lockdown	15.01 - 15.50	4.63 - 4.80	3.43 - 3.59	5.83 - 6.05	2.42 - 2.52
		Co-lockdown	7.25 - 7.43	2.64 - 2.70	1.78 - 1.81	3.20 - 3.28	1.37 - 1.41
		Pre-Co Difference	7.65 - 8.18	1.95 - 2.14	1.64 - 1.81	2.58 - 2.81	1.03 - 1.13
DWK	Tertiary Sediments	Pre-lockdown	9.83 - 10.30	0.64 - 0.67	0.23 - 0.24	0.69 - 0.72	0.097-0.102
		Co-lockdown	8.79 - 9.02	0.49 - 0.51	0.11 - 0.12	0.50 - 0.53	0.052-0.053
		Pre-Co Difference	0.89 - 1.41	0.13 - 0.18	0.12 - 0.13	0.17 - 0.21	0.045-0.050
MOR	Deccan Traps	Pre-lockdown	2.39 - 2.47	0.81 - 0.83	1.55 - 1.58	1.77 - 1.81	0.55 - 0.57
		Co-lockdown	0.65 - 0.69	0.15 - 0.16	0.36 - 0.38	0.39 - 0.41	0.24 - 0.26
		Pre-Co Difference	1.71 - 1.80	0.65 - 0.67	1.18 - 1.21	1.36 - 1.40	0.30 - 0.32
JUN	Deccan Traps	Pre-lockdown	1.47 - 1.67	0.29 - 0.32	0.63 - 0.67	0.70 - 0.74	0.85 - 0.90
		Co-lockdown	1.28 - 1.39	0.18 - 0.20	0.36 - 0.38	0.40 - 0.43	0.58 - 0.63
		Pre-Co Difference	0.17 - 0.29	0.10 - 0.13	0.26 - 0.30	0.28 - 0.33	0.24 - 0.30
LAL	Deccan Traps	Pre-lockdown	0.84 - 0.88	0.37 - 0.39	0.69 - 0.73	0.79 - 0.83	0.90 - 0.94
		Co-lockdown	0.70 - 0.73	0.27 - 0.29	0.52 - 0.54	0.59 - 0.62	0.63 - 0.65
		Pre-Co Difference	0.12 - 0.17	0.09 - 0.11	0.15 - 0.19	0.18 - 0.22	0.26 - 0.31
UKI	Deccan Traps	Pre-lockdown	1.44 - 1.48	0.65 - 0.66	0.26 - 0.27	0.70 - 0.72	0.12 - 0.13
		Co-lockdown	0.89 - 1.06	0.42 - 0.44	0.138 - 0.142	0.45 - 0.46	0.080-0.082
		Pre-Co Difference	0.42 - 0.48	0.21 - 0.24	0.12 - 0.13	0.26 - 0.27	0.043-0.046
UNA	Deccan Traps	Pre-lockdown	0.437 - 0.496	0.046 - 0.051	0.023 - 0.026	0.053 - 0.058	0.015-0.016
		Co-lockdown	0.422 - 0.453	0.036 - 0.038	0.025 - 0.026	0.045 - 0.047	0.014-0.016
		Pre-Co Difference	0.010-0.054	0.009-0.014	0.001-0.004	0.007-0.012	0.001-0.003
SUR	Cretaceous Rocks	Pre-lockdown	0.86 - 0.92	0.41 - 0.45	0.19 - 0.20	0.46 - 0.50	0.17 - 0.18
		Co-lockdown	0.61 - 0.65	0.31 - 0.33	0.15 - 0.16	0.35 - 0.37	0.157-0.160
		Pre-Co Difference	0.22 - 0.27	0.10 - 0.14	0.040 - 0.044	0.103 - 0.144	0.013-0.017
BDR	Jurassic Rocks	Pre-lockdown	2.02 - 2.34	0.65 - 0.71	0.68 - 0.73	0.96 - 1.04	0.43 - 0.45
		Co-lockdown	1.44 - 1.58	0.51 - 0.56	0.49 - 0.53	0.73 - 0.79	0.36 - 0.37
		Pre-Co Difference	0.50 - 0.84	0.10 - 0.16	0.16 - 0.22	0.20 - 0.27	0.06 - 0.09
GDD	Jurassic Rocks	Pre-lockdown	0.94 - 1.09	0.26 - 0.28	0.32 - 0.34	0.41 - 0.44	0.37 - 0.40
		Co-lockdown	0.90 - 0.97	0.25 - 0.27	0.22 - 0.24	0.35 - 0.37	0.28 - 0.30
		Pre-Co Difference	0.005 - 0.014	0.004 - 0.009	0.08 - 0.11	0.05 - 0.09	0.08 - 0.11
SIP	Proterozoic Rocks	Pre-lockdown	0.58 - 0.62	0.137 - 0.143	0.138 - 0.147	0.197 - 0.207	0.108-0.114
		Co-lockdown	0.46 - 0.61	0.096 - 0.105	0.095 - 0.103	0.138 - 0.15	0.073-0.078

		Pre-Co Difference	0.02 - 0.13	0.034 - 0.043	0.037 - 0.046	0.053 - 0.061	0.034 - 0.04

Table S3: Range of the estimated population mean of seismic noise levels in d_{rms} (in nm) for nighttime with 95% confidence level in different frequency ranges, for Pre- and Co-lockdown periods. The range is also estimated for the population mean observed in difference in the d_{rms} estimates between Pre- and Co-lockdown periods.

Stns.	Geology	Duration	Frequency Range				
			1 - 5 Hz	5 - 10 Hz	10 - 15 Hz	5 - 15 Hz	15 - 20 Hz
BHI	Quaternary Sediments	Pre-lockdown	17.08 - 18.06	1.27 - 1.36	1.43 - 1.54	0.59 - 0.63	0.51 - 0.55
		Co-lockdown	11.27 - 12.00	0.90 - 0.98	1.02 - 1.09	0.42 - 0.46	0.34 - 0.37
		Pre-Co Difference	5.34 - 6.58	0.32 - 0.45	0.36 - 0.49	0.15 - 0.20	0.15 - 0.20
RAI	Quaternary Sediments	Pre-lockdown	12.08 - 12.43	1.94 - 2.03	2.26 - 2.38	1.10 - 1.21	0.75 - 0.83
		Co-lockdown	6.80 - 7.00	1.44 - 1.51	1.68 - 1.76	0.84 - 0.88	0.71 - 0.75
		Pre-Co Difference	5.15 - 5.48	0.45 - 0.58	0.52 - 0.65	0.24 - 0.32	0.03 - 0.09
DWK	Tertiary Sediments	Pre-lockdown	10.48 - 11.03	0.12 - 0.14	0.13 - 0.14	0.04 - 0.05	0.02 - 0.03
		Co-lockdown	9.96 - 10.13	0.12 - 0.14	0.13 - 0.14	0.03 - 0.04	0.024-0.025
		Pre-Co Difference	0.47 - 1.08	0.003 - 0.007	0.003 - 0.008	0.006 - 0.011	0.002-0.005
MOR	Deccan Traps	Pre-lockdown	1.64 - 1.69	0.69 - 0.70	1.30 - 1.36	1.12 - 1.15	0.32 - 0.33
		Co-lockdown	0.58 - 0.60	0.10 - 0.11	0.20 - 0.22	0.17 - 0.19	0.10 - 0.11
		Pre-Co Difference	1.05 - 1.11	0.59 - 0.60	1.12 - 1.15	0.94 - 0.97	0.21 - 0.22
JUN	Deccan Traps	Pre-lockdown	1.25 - 1.40	0.13 - 0.14	0.30 - 0.20	0.27 - 0.28	0.44 - 0.46
		Co-lockdown	1.15 - 1.18	0.12 - 0.13	0.229 - 0.241	0.19 - 0.201	0.54 - 0.56
		Pre-Co Difference	0.08 - 0.23	0.012 - 0.017	0.07 - 0.09	0.07 - 0.09	(-0.11) - (-0.08)
LAL	Deccan Traps	Pre-lockdown	0.73 - 0.78	0.31 - 0.32	0.55 - 0.58	0.45 - 0.48	0.65 - 0.69
		Co-lockdown	0.66 - 0.68	0.22 - 0.23	0.42 - 0.44	0.35 - 0.37	0.39 - 0.42
		Pre-Co Difference	0.06 - 0.10	0.08 - 0.10	0.12 - 0.16	0.09 - 0.12	0.24 - 0.28
UKI	Deccan Traps	Pre-lockdown	1.17 - 1.22	0.48 - 0.50	0.52 - 0.54	0.186 - 0.19	0.088 - 0.09
		Co-lockdown	0.87 - 0.95	0.38 - 0.41	0.39 - 0.42	0.11 - 0.12	0.055-0.060
		Pre-Co Difference	0.24 - 0.33	0.08 - 0.11	0.10 - 0.14	0.07 - 0.08	0.030-0.035
UNA	Deccan Traps	Pre-lockdown	0.40 - 0.43	0.024-0.026	0.030-0.033	0.016-0.018	0.010-0.012
		Co-lockdown	0.36 - 0.37	0.030 - 0.033	0.036 - 0.039	0.018 - 0.020	0.013-0.014
		Pre-Co Difference	0.04 - 0.06	(-0.008) – (-0.003)	(-0.008) – (-0.004)	(-0.003) – (-0.001)	(-0.004) – (-0.003)
SUR	Cretaceous Rocks	Pre-lockdown	0.79 - 0.82	0.33 - 0.36	0.35 - 0.39	0.12 - 0.13	0.10 - 0.11
		Co-lockdown	0.60 - 0.62	0.31 - 0.34	0.33 - 0.36	0.10 - 0.11	0.10 - 0.11
		Pre-Co Difference	0.18 - 0.21	0.01 - 0.03	0.01 - 0.04	0.01 - 0.02	0.001-0.003
BDR	Jurassic Rocks	Pre-lockdown	1.77 - 1.92	0.28 - 0.32	0.41 - 0.46	0.29 - 0.32	0.30 - 0.31
		Co-lockdown	1.07 - 1.14	0.25 - 0.27	0.36 - 0.39	0.26 - 0.28	0.28 - 0.29
		Pre-Co Difference	0.66 - 0.78	0.02 - 0.06	0.05 - 0.08	0.02 - 0.06	0.01 - 0.03
GDD	Jurassic Rocks	Pre-lockdown	0.61 - 0.72	0.07 - 0.09	0.12 - 0.14	0.09 - 0.11	0.14 - 0.16
		Co-lockdown	0.59 - 0.66	0.08-0.09	0.14 - 0.16	0.11 - 0.13	0.20 - 0.24
		Pre-Co Difference	0.02 - 0.87	(-0.01) – (0.005)	(-0.03) – (-0.005)	(-0.03) – (-0.01)	(-0.09) – (-0.05)
SIP		Pre-lockdown	0.43 - 0.58	0.078 - 0.081	0.113 - 0.118	0.080 - 0.085	0.061-0.064

	Proterozoic Rocks	Co-lockdown	0.43 - 0.54	0.057 - 0.069	0.074 - 0.087	0.045 - 0.05	0.037 - 0.04
		Pre-Co Difference	0.01 - 0.07	0.014 - 0.023	0.028 - 0.042	0.032 - 0.038	0.022-0.026
